# Understanding stability and reactivity of transition metal single-atoms on graphene

**DOI:** 10.1038/s41598-025-00126-y

**Published:** 2025-05-03

**Authors:** Wesley Oliveira Morais, João Paulo Cerqueira Felix, Gabriel Reynald da Silva, Carlos Maciel de Oliveira Bastos, Alexandre C. Dias, Efracio Mamani Flores, Celso R. C. Rêgo, Vinícius da Silva Ramos de Sousa, Diego Guedes-Sobrinho, Maurício J. Piotrowski

**Affiliations:** 1 Department of Physics, Federal University of Pelotas, Pelotas, 96010-900 Brazil; 2 Institute of Physics Armando Dias Tavares, Rio de Janeiro State University, Rio de Janeiro, 20550-900 Brazil; 3 Chemistry Department, Federal University of Paraná, Curitiba, 81531-980 Brazil; 4 Institute of Physics and International Center of Physics, University of Brasília, Brasília, 70919-970 Brazil; 5 Department of Physics, Jorge Basadre Grohmann National University, Tacna, 23000 Peru; 6 Institute of Nanotechnology Hermann-von-Helmholtz-Platz, Karlsruhe Institute of Technology, Karlsruhe, 76021 Germany

**Keywords:** Single-atom catalysts, Density functional theory, Adsorption, Nanocatalysis, Transition-metals, Substrates, van der Waals, Chemistry, Energy science and technology, Materials science, Nanoscience and technology

## Abstract

Recently, single-atom catalysts (SACs) based on transition metals (TMs) have been identified as highly active catalysts with excellent atomic efficiency, reduced consumption of expensive materials, well-defined active centers, and tunable activity and selectivity. Furthermore, when carbon-based supports (including graphene-derived materials) are employed in SACs, their unique structural and electronic properties, such as high electrical conductivity and mechanical strength, can be integrated. However, for this application, the primary objective is to maintain proper stability-reactivity balance, ensuring the system remains stable while preserving its high chemical activity. In this context, we explore the adsorption behavior of TM single atoms (Co, Ni, Rh, Pd, Ir, Pt) on pristine graphene (pGR), hexagonal boron nitride (hBN), and graphene with monovacancies (GRm) using DFT-PBE+D3 calculations. From the adsorption energy trends, we observe weak chemisorption on pGR and physisorption on hBN, with adsorption energies ranging from 0.5 eV (Co/hBN) to 1.80 eV (Rh/pGR). In contrast, the adsorption strength is significantly enhanced on GRm (strong chemisorption), with adsorption energies reaching up to 9.11 eV for Ir/GRm, attributed to the strong defect-induced reactivity and improved orbital overlap. Electronic structure analysis reveals that pGR retains its semimetallic nature, hBN remains an insulator, and GRm transitions to metallic behavior due to the strong interactions between TM-C. Bader charge analysis indicates significant charge transfer in GRm, consistent with its catalytic potential, while hybridization indices show substantial *pd* orbital mixing, favoring improved TM anchoring. Thus, our results identify GRm as the most promising substrate for SACs, pGR as a balanced platform for controlled reactivity, and hBN as a stable support for selective catalysis or dielectric applications. Finally, defect engineering is a powerful strategy for designing next-generation catalysts, ensuring the right balance between stability and reactivity.

## Introduction

Single-atom catalysts (SACs) have caused a revolution in catalysis since they bridge the gap between homogeneous and heterogeneous approaches.^1–3^ SACs make the most of atomic efficiency, provide well-defined active sites (designed active centers), and allow for adjustable reactivity and outstanding selectivity.^4,5^ By spreading individual metal atoms on proper supports, they best use rare transition metals (TMs) while allowing in-depth studies of catalytic mechanisms.^1,2^ Yet, keeping single metal atoms stable is tough. Their high surface energy promotes aggregation into clusters or nanoparticles, which hurts their catalytic activity.^3,6^ To mitigate this, 2D supports offer stable anchoring sites while balancing stability and reactivity.^5^

The pioneering work of Qiao et al. on $\text {Pt}_1$/$\text {FeO}_x$ demonstrated how iron oxide-supported single Pt atoms exhibit remarkable catalytic activity and stability, particularly for CO oxidation, due to partially vacant Pt-5*d* orbitals that enhance reactant interactions.^4^ Since then, SACs have become an important focus of research with applications in electrocatalysis,^6–8^ pollutant degradation,^9,10^ and energy.^11,12^ Their innovation lies in atomic dispersion on supports, minimizing the use of expensive noble metals, and ensuring full and efficient metal utilization while imparting unique electronic and structural properties to SACs that differentiate them from traditional nanocatalysts,^5^ maximizing their catalytic efficiency.^3,13^ The low-coordination environment of single atoms enhances the catalytic activity and selectivity for key reactions, including CO_2_ reduction, oxidation, water splitting, and nitrogen fixation.^3,4,6^

Among the possible SAC supports, carbon-based 2D materials (graphene-derived systems) stand out due to their unique structure and electronics.^14,15^ Pristine graphene (pGR) offers excellent electrical conductivity, mechanical robustness, and tunable surface chemistry,^16,17^ and is considered a promising and malleable platform to stabilize individual metal atoms and facilitate efficient charge transfer. However, the catalytic performance and, thus, its stability are limited by the weak interaction with noble metals (e.g., Au).^1,5^ To address this, the metal–graphene bond and the catalytic characteristics have been significantly improved by alloying strategies that incorporate TMs (e.g., Ni, Pd, or Pt).^18^ Moreover, functionalization by monovacancies and/or doping is an alternative that can produce localized defect states that strengthen metal-support interactions while preventing metal aggregation, a significant problem in SAC design.^13,19–22^ For selective catalysis and/or applications requiring minimal substrate alteration, other 2D materials, like hexagonal boron nitride (hBN), can offer inert supports,^23–25^ being focus of additional research.

Among the possible SAC TMs, Co, Ni, Rh, Pd, Ir, and Pt have been most sought after based on their catalytic characteristics, electronic attributes, and experimental accessibility.^26–28^ Pt, Ir, and Rh are very good catalysts but are very expensive, hence perfect benchmarking references for performance. By comparison, Co and Ni are cheaper and more accessible, with the potential for future alternatives in catalytic use. Pd is a compromise, balancing between catalysis activity and cost. They show significant activity in many hydrogenation, oxidation, and C-C coupling reactions. They have been well explored under homogeneous and heterogeneous catalysis due to their stability and versatility. Aside from their catalytic use, these metals exhibit stable complexes with common substrates, such as carbon and oxygen-containing compounds, and are model systems for investigating metal-support interactions.^4^ They electronically span from fairly electropositive to extremely electronegative species and are well suited to dissecting electronic structure effects upon catalytic performance, bonding character, charge transfer, and activation energy. Their *d*-electron population, atomic size, and electronegativity significantly influence the catalytic activity, making them good candidates to be studied. Experimental studies also show the role of metal-support interactions in regulating catalytic efficiency. For instance, Pd-supported catalysts over reduced graphene oxide (RGO) have shown enhanced electrocatalytic activity towards alcohol oxidation through synergistic interactions between the Pd and the graphene support.^29^ Besides, Pt and Ir nanoparticle dispersion in graphene has been demonstrated to exhibit improved catalytic activity in ammonia oxidation reactions via effective metal nanoparticle dispersion.^30^ Also, Ni and Co alloying with graphene has been proven to design the electronic structure of Pt-supported catalysts, thereby enhancing the catalytic activity in CO oxidation reactions.^31^ All these findings demonstrate the importance of graphene-based supports for designing the electronic properties of TMs and thus optimizing their catalytic activities.

The atomic-scale interactions in SACs have been mainly clarified by theoretical-computational investigations, particularly those that rely on density functional theory (DFT).^5,32^ These studies aim to identify the effects of metal–support interactions, quantum confinement, and defect-induced reactivity to guide experimental efforts in SAC design.^9,33^ Computational studies have also demonstrated the importance of integrating theoretical and experimental approaches (in synergy) and the relevance of substrate engineering in adjusting electronic structures and catalytic characteristics.^19^

Even with advances, we still need to figure out how to balance stability and reactivity. Although pGR offers structural stability, its low reactivity reduces catalytic performance. Conversely, monovacancy graphene (GRm) generated reactive defect sites that improved TM anchoring and resulted in significant electrical changes.^34,35^ Homeostasis must be attained to preserve SAC lifetime and activity. Electroreduction of CO_2_ and methane activation have been successful examples of graphene functionalization in terms of catalytic characteristics. This is because graphene functionalization increases single-atom reactivity and leverages substrate stability.^13^ To better understand the connection between substrate modification, TM selection, and the possibilities of other materials (such as hBN), more research is needed.

In the present study, through DFT-based calculations with semi-empirical van der Waals corrections (Grimme’s D3), we investigated the single-atom (Co, Ni, Rh, Pd, Ir and Pt) adsorption behavior on graphene-derived substrates (pGR, GRm and hBN). By analyzing the adsorption energy and its decomposition, electronic structure changes, charge transfer, and hybridization indices trends, we elucidate the relationship between stability and reactivity in these systems. Our findings reveal distinct TM adsorption trends ranging from physisorption on hBN to strong chemisorption on GRm, highlighting the role of substrate engineering in SAC design. This study provides a possible roadmap for tuning SAC performance through defect engineering and material selection. While in the present work, stability and electronic properties of SACs under vacuum are probed, we realize stability in catalytic systems, to a large extent, is reaction environment-sensitive. For reporting trends in stability, adsorption energies are related to cohesive energies to quantify non-agglomeration resistance, and charge transfer is explored to highlight metal–support interaction. While direct catalytic simulations lie outside the remit of this paper, our conclusions regarding electronic structure changes, charge redistribution, and metal–support bonding provide a foundational understanding essential to understanding SAC reactivity in catalytic processes.

## Methods

### Computational details and theoretical approach

Our spin-polarized DFT calculations^36,37^ were performed using the Vienna *Ab Initio* Simulation Package (VASP)^38,39^ to solve the Kohn–Sham (KS) equations. We used the generalized gradient approximation (GGA) via the Perdew–Burke–Ernzerhof (PBE) functional^40^ to account for exchange and correlation effects. Long-range dispersion interactions, van der Waals (vdW) corrections, were incorporated via Grimme’s semi-empirical D3 method,^41–44^ which is particularly relevant for TM–2D material interactions. Our calculation protocol: DFT-PBE+D3 is reliable in previous modeling of adsorption phenomena in graphene-based systems.^45–49^

For the expansion of the KS orbitals, we used a set of plane wave basis functions with an energy cutoff of 600 eV. The core-valence interactions were described employing the projector augmented wave (PAW) method,^50,51^ employing a fully relativistic treatment for core electrons and a scalar-relativistic approximation for valence electrons.^52,53^ We used a Gaussian smearing with a width of 1 meV to improve the electron convergence and stabilize the occupancies near the Fermi level.

The examined substrates (pGR, GRm, and hBN) were modeled using a $6\times 6\times 1$ supercell in the *xy* plane, ensuring negligible interaction between periodic images. The supercells contained 72 atoms for pGR (C atoms) and hBN (36 B and 36 N atoms), while the GRm system comprised 71 C atoms to represent a (planar) monovacancy. Transition metal (Co, Ni, Rh, Pd, Ir, Pt) adsorption was examined at the top, bridge, hollow, and substitutional (vacancy) sites to comprehensively assess the TM–2D material interactions. A vacuum layer of 17 Å along the *z*-axis was included to eliminate spurious interlayer effects.

For the periodic system calculations, the Brillouin zone was sampled using a $4\times 4\times 1$ $\textbf{k}$-point mesh. In contrast, the free-atom and dimer calculations were conducted on the $\Gamma$-point within a large orthorhombic and cubic box, respectively, to avoid periodic image interactions. For bulk calculations, we used a $\textbf{k}$-mesh of $22\times 22\times 22$. We performed the structural optimizations using as convergence criteria the values of 0.015eV/Å for Hellmann-Feynman forces and 1.0 × 10^–6^ eV for electronic self-consistency, ensuring that all configurations corresponded to stable structures. For TM bulk systems, we obtained the equilibrium volumes by stress tensor optimization and atomic forces using a cutoff energy two times larger than the recommended values. It is important to emphasize that all systems studied here were obtained from spin-polarized calculations, consistently treating the electronic structure and magnetism of magnetic and non-magnetic transition metals. Detailed convergence tests for supercell size, cutoff energy, and $\textbf{k}$-point sampling are provided in the Supporting Information (Tables S1−S7).

### Analyses

#### Energy analysis

We used multiple energy metrics to evaluate TM adatoms’ stability and interaction properties on substrates. In addition to the widely used relative total energy, we assessed the energy stability of the systems under study using the binding energy ($E_{\text {b}}$), which is defined as follows:

1 $$\begin{aligned} E_{\text {b}} = \frac{E_{\text {tot}}^{{sub}} - nE_{\text {tot}}^{\text {free-atom}}}{n}~, \end{aligned}$$

where $E_{\text {tot}}^{{sub}}$ and $E_{\text {tot}}^{\text {free-atom}}$ denote the total energies of the substrate (sub = pGR, GRm, hBN) and a free constituent atom, respectively. A more (less) negative $E_{\text {b}}$ value indicates a more (less) stable arrangement. Similarly, we have calculated the binding/cohesive energy ($E_{\text {2b/coh}}$) for the TM dimers and bulk systems by the following equation:

2 $$\begin{aligned} E_{\text {2b/coh}} = \frac{E_{\text {tot}}^{\text {TM}_{2}/bulk} - nE_{\text {tot}}^{\text {free-atom}}}{n}~, \end{aligned}$$

where $E_{\text {tot}}^{\text {TM}_{2}/bulk}$ and $E_{\text {tot}}^{\text {free-atom}}$ denote the total energies of the TM dimer/bulk and free-atoms, respectively, for TM $=$ Co, Ni, Rh, Pd, Ir, and Pt.

The monovacancy formation energy ($E_{\text {vac}}$) constituting the GRm case was calculated as follows:

3 $$\begin{aligned} E_{\text {vac}} = E_{\text {tot}}^{\text {GRm}} - \frac{(n-1)E_{\text {tot}}^{\text {pGR}}}{n}~, \end{aligned}$$

where $E_{\text {tot}}^{\text {GRm}}$ and $E_{\text {tot}}^{\text {pGR}}$ represent the total energies of GRm and pGR, respectively. $E_{\text {vac}}$ measures the energy required to remove a C atom from pGR, creating a monovacancy.

The adsorption energy ($E_{\text {ads}}$) was obtained as follow:

4 $$\begin{aligned} E_{\text {ads}} = E_{\text {tot}}^{{TM{\text {/}}sub}} - E_{\text {tot}}^{\text {TM free-atom}} - E_{\text {tot}}^{{sub}}~, \end{aligned}$$

where $E_{\text {tot}}^{{TM{\text {/}}sub}}$ is the total energy of the TM/sub system. $E_{\text {ads}}$ provides the energy required to remove a TM adatom from its substrate.

We calculated the interaction energy ($\Delta E_{\text {int}}$) and substrate distortion energy ($\Delta E_{\text {dis}}$) to separate adsorption effects:

5 $$\begin{aligned} \Delta E_{\text {int}} = E_{\text {tot}}^{{TM{\text {/}}sub}} - E_{\text {tot}}^{{sub}~\text {frozen}} - E_{\text {tot}}^{\text {TM free-atom}}~, \end{aligned}$$

and

6 $$\begin{aligned} \Delta E_{\text {dis}} = \frac{E_{\text {tot}}^{{sub}~\text {frozen}} - E_{\text {tot}}^{{sub}}}{n}~, \end{aligned}$$

where $E_{\text {tot}}^{{sub}~\text {frozen}}$ is the substrate’s total energy in the TM/sub system in its frozen geometry. Since $\Delta E_{\text {dis}}$ accounts for the effect of substrate distortions brought on by interaction with TMs, $\Delta E_{\text {int}}$ represents the energy gain from adsorption without taking this effect into account. After that, the adsorption energy may be written as follows:^45–48,54,55^

7 $$\begin{aligned} E_{\text {ads}} = \Delta E_{\text {int}} + n{\Delta }E_{\text {dis}}~. \end{aligned}$$

#### Structural analysis

We used the effective coordination number (ECN)^56,57^ framework and VESTA^58^ to characterize TM–substrate bonding. Average bond lengths ($d_{\text {av}}$), TM–substrate distances ($d_{\text {TM-sub}}$), ECN, and their relative changes following TM adsorption ($\Delta d_{\text {av}}$ and ${\Delta }\text {ECN}$) were among the structural parameters that were examined. Specifically, $\Delta d_{\text {av}}$ and ${\Delta }\text {ECN}$ are obtained as follow:

8 $$\begin{aligned} {\Delta }d_{\text {av}} = \frac{\left( d_{\text {av,ads}} - d_{\text {av}} \right) {\times }100}{d_{\text {av}}}~, \end{aligned}$$

and

9 $$\begin{aligned} {\Delta }\text {ECN} = \frac{\left( \text {ECN}_{\text {ads}} - \text {ECN}\right) {\times }100}{\text {ECN}}~. \end{aligned}$$

#### Electronic structure and bonding analysis

We used the total magnetic moment ($m_{\text {T}}$), the density of states (DOS), crystal orbital Hamilton population (COHP), Bader charge analysis, and hybridization index computation, to determine the electronic and magnetic characteristics of the systems. The Local-Orbital Basis Suite Towards Electronic-Structure Reconstruction (LOBSTER) was used to calculate DOS and COHP.^59^ The electronic structure is visualized by the DOS, which displays the number of states accessible at each energy relative to the Fermi energy. Peaks near the Fermi level indicate potential electron occupancy or transfer channels, highlighting chemically active orbitals.

We used COHP analysis, specifically the projected COHP (pCOHP), which breaks down band structure energy into bonding, nonbonding, and antibonding contributions, to examine electronic interactions further.^60^ This approach assesses overlap populations and the Hamiltonian’s off-site components (hopping terms). It is possible to distinguish between bonding contributions because antibonding interactions show negative pCOHP values, whereas bonding interactions correspond to positive overlap populations and negative Hamiltonian elements.

We conducted Bader charge analysis to obtain the charge distribution and transfer analysis,^61^ which, under the following condition, divides charge density into atomic regions known as Bader volumes ($V_{\text {Bader}}$), defined by zero-flux surfaces ($S(\textbf{r}_{s})$): $\nabla n(\textbf{r}_{s}) \cdot S(\textbf{r}_{s}) = 0$.^62^ For an atomic site $\alpha$ within a Bader volume ($V_{\text {Bader}} = V_{\alpha ,s}$), the effective charge of an atom is determined as follows:

10 $$\begin{aligned} \Delta Q_{\alpha }^{\text {Bader}} = Z_{\alpha } - \int _{V_{\alpha }} n(\textbf{r}) d^{3}r~, \end{aligned}$$

where $Z_{\alpha }$ is the atomic valence, and the integral represents the total electronic population within the Bader volume. Considering $\Delta Q_{\text {eff}} = \Delta Q_{\alpha }^{\text {Bader}}$, a positive ($\Delta Q_{\text {eff}} > 0$) or negative ($\Delta Q_{\text {eff}} < 0$) net charge means cationic or anionic behavior, respectively. This charge determination approach is optimized through topological methods such as Voronoi polyhedra,^63^, and it provides a framework for understanding charge transfer and distribution throughout the structure.

Finally, we examined the hybridization index (*hyb*)^64^ to characterize orbital interactions. This index is derived from the sum of the direct product of local densities of states (*s*, *p*, and *d*) in regions where all contributions are nonzero.^48,65,66^ It is calculated as follows:

11 $$\begin{aligned} hyb_{kl} = \sum _{I=1}^{N}\sum _{i=1}^{occ} w_{i,k}^{(I)}w_{i,l}^{(I)}~, \end{aligned}$$

where *k*, *l* correspond to *s*, *p*, or *d* states ($k \ne l$), and $w_{i,k}^{(I)}$, $w_{i,l}^{(I)}$ are projections of the *i*-th KS orbital onto spherical harmonics centered at atom *I*. This technique qualitatively estimates complex systems’ orbital mixing and bonding properties by detecting contributions from *sd*, *sp*, or *pd* hybridization.

## Results

### Individual systems

Determining the unit cell and assembling the supercell are the first steps in building each substrate. Graphene’s unit cell consists of two C atoms that form a hexagonal lattice with an interatomic distance of 1.42 Å and lattice parameters of 2.46 Å, in agreement with literature.^67^ This configuration produces a binding energy of -7.84 eV/atom and a zero total magnetic moment, which is in good agreement with earlier research.^45,68^

**Fig. 1:**
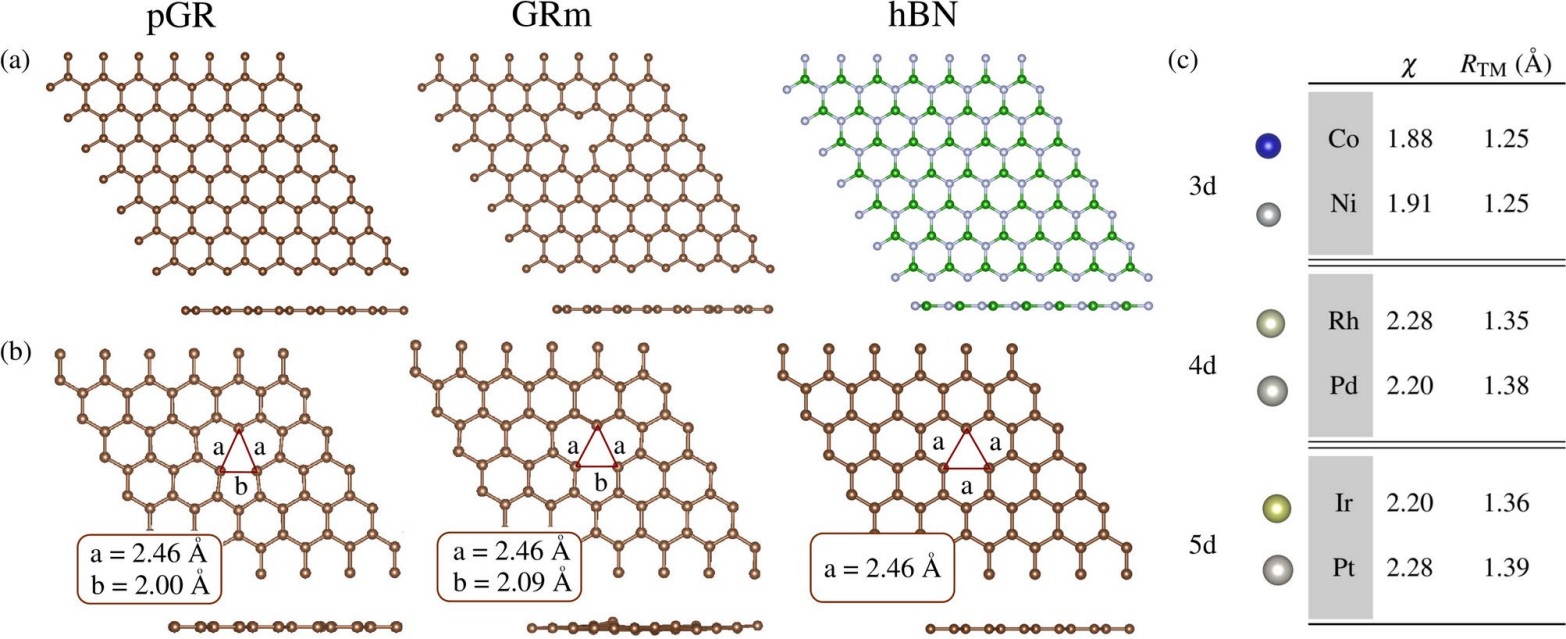
(**a**) Schematic representation (top and side views) of the substrates: pristine graphene (pGR), graphene with monovacancy (GRm), and hexagonal boron nitride (hBN). (**b**) Monovacancy models (top and side views): non-symmetric planar, non-symmetric non-planar, and symmetric planar, with corresponding C-C distances (*a* and *b*) around the monovacancy. (**c**) Representation of studied TMs from the 3*d* (Co and Ni), 4*d* (Rh and Pd), and 5*d* (Ir and Pt) TM series, along with their electronegativities ($\chi$)^70^ and atomic radii ($R_{\text {TM}}$).

The size of graphene supercells varies according to the study’s goals and the need for adsorption; this ensures enough space between periodic images to avoid interactions.^69^ For example, sufficient separation is required to prevent false interactions when simulating point defects such as monovacancy or adatom adsorption.^21^ For pGR, a $6\times 6\times 1$ supercell ensures minimal separation of 15 Å in the *xy* plane and 17 Å along the *z* axis. This arrangement maintains a binding energy close to that of the unit cell (-7.88 eV/atom), preserves an average C-C bond length of 1.42 Å, a coordination number of 3.0, and retains a zero total magnetic moment.^45,46^ Figure 1(a) shows a representation of the pGR substrate, as well as the GRm and hBN substrates, derived from convergence tests shown in the Supporting Information .

Following a similar procedure, the hBN unit cell consists of one B and one N atom, forming a honeycomb lattice with electronic properties analogous to graphene (BN pairs electronically equivalent to CC pairs). The hBN substrate, constructed with a $6\times 6\times 1$ supercell, exhibits a binding energy of -7.04 eV/atom, a B-N bond length of 1.45 Å, a coordination number of 3.0, and non-magnetic behavior, in agreement with the literature.^71,72^ Both pGR and hBN are highly stable and chemically inert in their pristine forms. Strong $sp^2$ C-C bonding and a delocalized $\pi$-electron system stabilize pGR while decreasing its reactivity, especially with nonpolar species that physisorb mainly.^45–47^ Similarly, hBN shows high stability and low reactivity, both related to the ionic nature of hBN’s B-N bonds, where B is somewhat electropositive and N is electronegative.

Nevertheless, adding defects, functionalizing surfaces, or adsorbing/doping with additional atoms can all increase reactivity. Defects in pGR cause the $\pi$-conjugated system to be disrupted, which increases chemical activity. In contrast, hBN is less reactive than functionalized graphene even after modification because of its high bond energy and polarity. A common strategy for enhancing pGR reactivity is introducing monovacancies, forming GRm.^20,73,74^ This alteration unpairs electrons, increasing the magnetic moment from 0 ${\upmu _\text {B}}$ in pGR to 1.0-2.0 ${\upmu _\text {B}}$ in GRm. In monovacancies, distances among the three neighboring C atoms may change, consequently affecting the symmetry and planarity, originating different monovacancy configurations, as shown in Figure 1(b).

Upon optimization, GRm exhibits three dangling bonds around the vacancy. Its stable configuration consists of a symmetric, planar structure with equal C-C distances forming an equilateral triangle (2.46 Å). However, alternative metastable structures exist, including a non-symmetric planar configuration ($m_{\text {T}} = {1.5}\,{\upmu _\text {B}}$, distances of $a = {2.46}$ Å and $b = {2.00}$ Å), and a non-symmetric non-planar configuration (non-magnetic, distances of $a = {2.46}$ Å and $b = {2.09}$ Å), as shown in Figure 1(b).^21,75,76^ Initially, the geometric optimization leads to a metastable non-planar structure, where one C atom is displaced by 0.52 Å out of the *xy* plane, before forming a planar, non-symmetric monovacancy with two C atoms shifted closer to the vacancy, creating an isosceles triangle. This final planar non-symmetric structure has a binding energy of -7.77 eV/atom, a vacancy formation energy of -7.73 eV, and $m_{\text {T}} = {1.5}\,{\upmu _\text {B}}$, in good agreement with prior works.^21,45,46^ Finally, GRm is thus less stable than pGR by 0.11 eV, with a reduced coordination number of 2.95, and an increased average C-C bond length of 1.43 Å.

Stable substrates and reactive components must be combined for catalytic applications, especially in SACs. A promising balance between stability and reactivity is provided by the combination of TMs with pGR, hBN, and GRm. As seen in Figure 1(c), we picked representative TMs from the 3*d* (Co, Ni), 4*d* (Rh, Pd), and 5*d* (Ir, Pt) series. These were chosen based on their electronegativities ($\chi$) and atomic radii ($R_{\text {TM}}$). These TMs from groups 9 and 10 are extensively researched in theory and experiment because of their many catalytic uses and unfilled *d* orbitals^69,77–82^. The goal is to integrate the stability of graphene-based 2D substrates with the high reactivity of TM single-atoms, offering alternative configurations for SACs.

### Combined systems

#### Adsorption properties

**Fig. 2:**
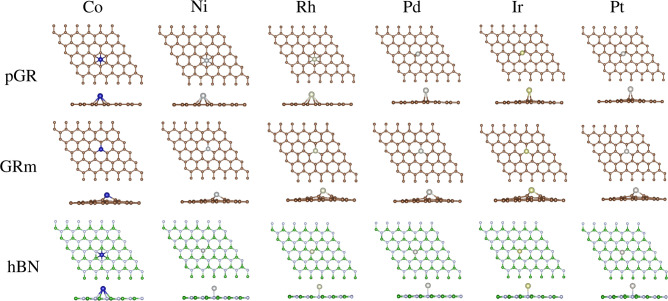
The lowest energy adsorbed configurations for the TM/sub systems (top and side views), where TM $=$ Co, Ni, Rh, Pd, Ir, and Pt; and sub $=$ pGR, GRm, and hBN.

The adsorption of TM atoms on graphene-based substrates is critical for the SACs design and advancing our understanding of heterogeneous catalysis. Such interactions are essential to prevent diffusion or agglomeration of metals, which could lead to nanoclusters or nanoparticle formation. Consequently, TMs were adsorbed at every potential non-equivalent sites on the substrates under study. These sites include hollow (above the hexagonal center), bridge (above a C-C bond), and top (above a C atom) for pGR. Similar sites exist for hBN, but there are two potential top sites: above N or B. For GRm, an extra adsorption site near the vacancy region was included, leading to an embedded configuration. Figure 2 illustrates the most stable TM/sub configurations, identifying the lowest energy adsorption sites, with before and after optimization configurations provided in the Supporting Information (Figures S1−S3). The main properties ($E_{\text {ads}}$, $d_{\text {TM-sub}}$, and $m_{\text {T}}$) resulting from TM adsorption on the most stable systems are presented in Table 1, while the binding energy for TM dimers ($E_{\text {2b}}$) and cohesive energy for TM bulk ($E_{\text {coh}}$) are presented in Table 2. In contrast, supplementary graphs and tables are provided in the Supporting Information (Figure S4 and Tables S8 and S9), detailing these properties for additional configurations, including higher-energy setups.

Figure 2 indicates that the most stable TM/pGR adsorption sites are hollow for Co, Ni, and Rh, while the bridge site is preferred for Pd, Ir, and Pt. This shift from hollow to bridge reflects a transition from open-shell to noble-metal behavior as *d*-state occupancy increases, which is aligned with the tendency to favor metal binding at sites offering optimal overlap between the metal’s *d*-orbitals and the $\pi$-system of graphene. In TM/GRm systems, the embedded site consistently emerges as the most stable configuration, enhancing binding strength (“anchoring”) and reaching -9.11 eV for Ir. GRm modifications facilitate stronger bonding by altering the local electronic structure and introducing defect states. For TM/hBN, the $\text {top}^{{N}}$ site is generally preferred, except for Co/hBN, where the hollow site is slightly more stable. The slight energy difference ($\approx$ 0.07 eV) highlights the role of N atoms in facilitating TM adsorption, since N atoms are more electronegative than B, promoting a higher attraction of the TMs through their *d*-orbital overlap.

We have considered $E_{\text {ads}}$ as a crucial indicator of the interaction strength between TMs and substrates, these values are summarized in the Table 1, they transition from physisorption to chemisorption across different substrates. On pGR, $E_{\text {ads}}$ ranges from -1.05 eV for Co and Ir to -1.80 eV for Rh (-1.75 eV for Pt). Because of structural changes that promote improved binding, stronger interactions take place on GRm, with noticeably greater negative values, such as -9.11 eV for Ir. $E_{\text {ads}}$ values for TM/hBN range from -0.51 eV (Co) to -1.74 eV (Pt), and they are comparable to pGR. Structurally, we obtained that adsorption distances vary from 1.76 Å for Co/GRm to 2.41 Å for Co/hBN. Consequently, shorter distances on GRm indicate stronger bonding, while longer $d_{\text {TM-sub}}$ values on pGR and hBN reflect weaker interactions. An increase in atomic number correlates with longer adsorption distances and weaker adsorption energies due to larger atomic radii limiting planar integration.

**Table 1:** TM/sub adsorption properties: the lowest energy adsorption site, adsorption energy ($E_{\text {ads}}$), minimum TM–substrate distances ($d_{\text {TM-sub}}$), and total magnetic moment ($m_{\text {T}}$).

TM/sub	Site	$E_{\text {ads}}$ (eV)	$d_{\text {TM-sub}}$ (Å)	$m_{\text {T}}$ ($\upmu _\text {B}$)
Co/pGR	hollow	-1.05	2.10	1.00
Ni/pGR	hollow	-1.50	2.11	0.00
Rh/pGR	hollow	-1.80	2.29	0.00
Pd/pGR	bridge	-1.31	2.17	0.00
Ir/pGR	bridge	-1.05	2.07	1.00
Pt/pGR	bridge	-1.75	2.09	0.00
Co/GRm	embedded	-7.59	1.76	1.00
Ni/GRm	embedded	-6.77	1.79	0.00
Rh/GRm	embedded	-8.46	1.90	1.00
Pd/GRm	embedded	-5.48	1.94	0.00
Ir/GRm	embedded	-9.11	1.90	1.00
Pt/GRm	embedded	-7.39	1.93	0.00
Co/hBN	hollow	-0.51	2.41	3.00
Ni/hBN	$\text {top}^{{N}}$	-1.18	1.85	0.00
Rh/hBN	$\text {top}^{{N}}$	-1.42	2.11	1.00
Pd/hBN	$\text {top}^{{N}}$	-1.26	2.13	0.00
Ir/hBN	$\text {top}^{{N}}$	-0.85	2.03	1.00
Pt/hBN	$\text {top}^{{N}}$	-1.74	2.01	0.00

We observed that the total magnetic moments vary significantly, ranging from ${0.0}\,{\upmu _\text {B}}$ (e.g., Ni/pGR) to ${3.0}\,{\upmu _\text {B}}$ (Co/hBN). Lower $m_{\text {T}}$ values on pGR and GRm suggest quenching of TM magnetic moments by graphene’s delocalized $\pi$-electrons. Conversely, higher $m_{\text {T}}$ on hBN, particularly for Co, indicate less quenching due to weaker $\pi$-*d* interactions and reduced delocalization of the TM’s *d*-electrons. Thus, $m_{\text {T}}$ correlates with system reactivity, as it reflects the spin ordering of valence-shell electrons in the metal atoms, influencing the stability (via anchoring) and catalytic potential of SACs.

The nature of TM–substrate interactions depends on the substrate’s properties. For pGR, weak chemisorption occurs via hybridization between TM *d*-orbitals and graphene’s delocalized $\pi$-system, reflecting a balance between vdW forces and orbital overlap, yielding moderate adsorption energies (-1.05 eV to -1.80 eV) and distances around 2.1 Å to 2.3 Å. Physisorption is found for hBN, with vdW forces dominating and negligible charge transfer, particularly for late TMs, with longer distances (up to 2.4 Å) and lower $E_{\text {ads}}$ values (-0.51 eV to -0.85 eV). In contrast, GRm promotes strong chemisorption because of monovacancy-induced reactivity, which results in shorter adsorption distances (1.8 Å) and robust bonding with highly negative $E_{\text {ads}}$ values (-5.48 eV to -9.11 eV). This variation emphasizes how substrate change is essential for customizing TM adsorption behavior and creating effective SACs.

**Table 2:** The binding energy ($E_{\text {2b}}$) for TM dimers and the cohesive energy ($E_{\text {coh}}$) for TM bulk systems, where TM $=$ Co, Ni, Rh, Pd, Ir, and Pt.

TM	$E_{\text {2b}}$ (eV/atom)	$E_{\text {coh}}$ (eV/atom)
Co	-1.27	-4.99
Ni	-1.35	-4.76
Rh	-1.72	-5.75
Pd	-0.65	-3.76
Ir	-2.37	-7.27
Pt	-1.88	-5.37

In Table 2, we present the $E_{\text {2b}}$ for $\text {TM}_2$ dimers and the $E_{\text {coh}}$ for bulk TM systems to assess potential aggregation tendencies in contrast to SAC stability. These values can be directly compared with the adsorption energy ($E_{\text {ad}}$) reported in Table 1 for different substrates. For TM adatoms adsorbed on pGR and hBN substrates, we observe that $E_{\text {ad}}$ is lower in magnitude than both $E_{\text {2b}}$ and $E_{\text {coh}}$, indicating that these substrates do not strongly anchor metal atoms, making aggregation more likely when multiple TM atoms are present. In contrast to this, for TM adatoms on defected graphene (GRm), the adsorption energy is considerably greater than both $E_{\text {2b}}$ and $E_{\text {coh}}$, revealing a high tendency for single atom adsorption over clustering. This result highlights the critical role of defects in stabilizing SACs by suppressing metal clustering efficiently.

#### In-depth energy analysis

**Fig. 3:**
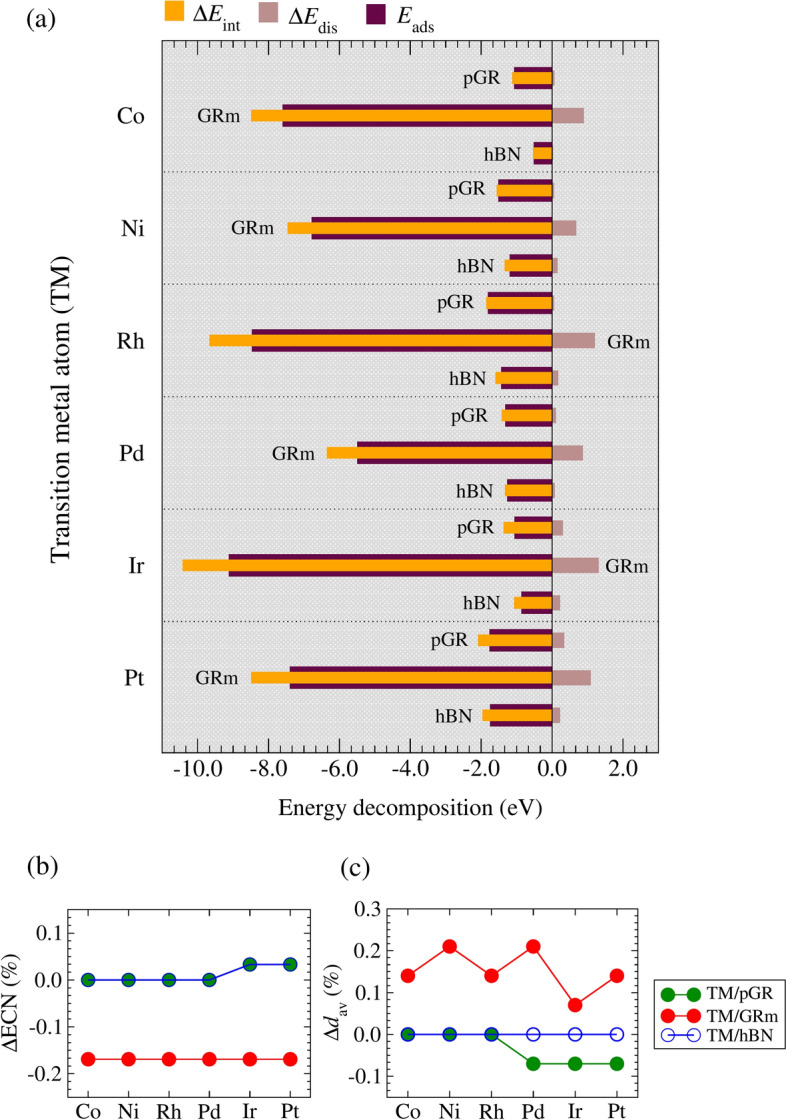
Decomposition of energy terms for the lowest energy TM/sub configurations, where TM = Co, Ni, Rh, Pd, Ir, and Pt; and sub = pGR, GRm, and hBN. (**a**) Adsorption energy ($E_\text {ads}$), interaction energy ($\Delta E_{\text {int}}$), and distortion energy ($\Delta E_{\text {dis}}$). (**b**) Relative deviation of the effective coordination number (${\Delta }\text {ECN}$). (**c**) The relative deviation of average bond lengths (${\Delta }d_\text {av}$) after TM adsorption.

To deepen our understanding from chemisorption (TM/GRm) to weak chemisorption (TM/pGR) and physisorption (TM/hBN) bonding situations, we decomposed the adsorption energy into its interaction and distortion energy-components. The associated structural modifications were analyzed by using changes in the ECN and $d_\text {av}$ after TM adsorption relative to the pristine substrates. These results are summarized in Figure 3, with panel (a) showing $E_\text {ads}$, $\Delta E_{\text {int}}$, and $\Delta E_{\text {dis}}$, while panels (b) and (c) present ${\Delta }\text {ECN}$ and ${\Delta }d_\text {av}$, respectively. Numerical values are provided in the Supporting Information (Table S10).

Across all substrates, interaction energy trends mirror those of adsorption energy. The GRm systems exhibit the most negative $\Delta E_{\text {int}}$, reaching -10.41 eV for Ir/GRm, due to the monovacancy-induced localized electronic states that enhance TM–substrate bonding. In contrast, the pGR and hBN systems show fewer negative values, indicative of weaker interactions dominated by surface effects rather than covalent bonding. Because of their advantageous *d*-orbital overlap, late TMs, like Pt and Rh, show more significant interactions for all substrate instances. As demonstrated in Ir/GRm with $\Delta E_{\text {dis}}$ of 1.30 eV, distortion energies are noticeably greater for GRm systems, indicating substantial structural rearrangements brought on by defect strain and TM bonding. However, $\Delta E_{\text {dis}}$ for pGR and hBN systems stays lower, suggesting that their pristine structures are not significantly disturbed.

The ECN values remain stable (constant) for pGR and hBN cases, averaging around 3.0, indicating minimal disruption to the local bonding environment. Consequently, ${\Delta }\text {ECN}$ is negligible for pGR and hBN. For GRm systems, a slight ECN decrease ($\approx$ 2.96) suggests partial reorganization due to TM embedding, with a slight negative ${\Delta }\text {ECN}$. The average bond lengths in pGR and hBN also show uniform bonding properties, staying relatively constant at 1.42 Å and 1.45 Å, respectively. Differently, with positive variations in ${\Delta }d_\text {av}$ ranging from 0.14% to 0.21%, GRm cases show slight elongation in dav (e.g., 1.425 Å for Ir/GRm). This elongation is a feature of chemisorption in GRm and is caused by TM-induced lattice strain, which weakens adjacent C-C bonds.

#### Electronic analysis

**Fig. 4:**
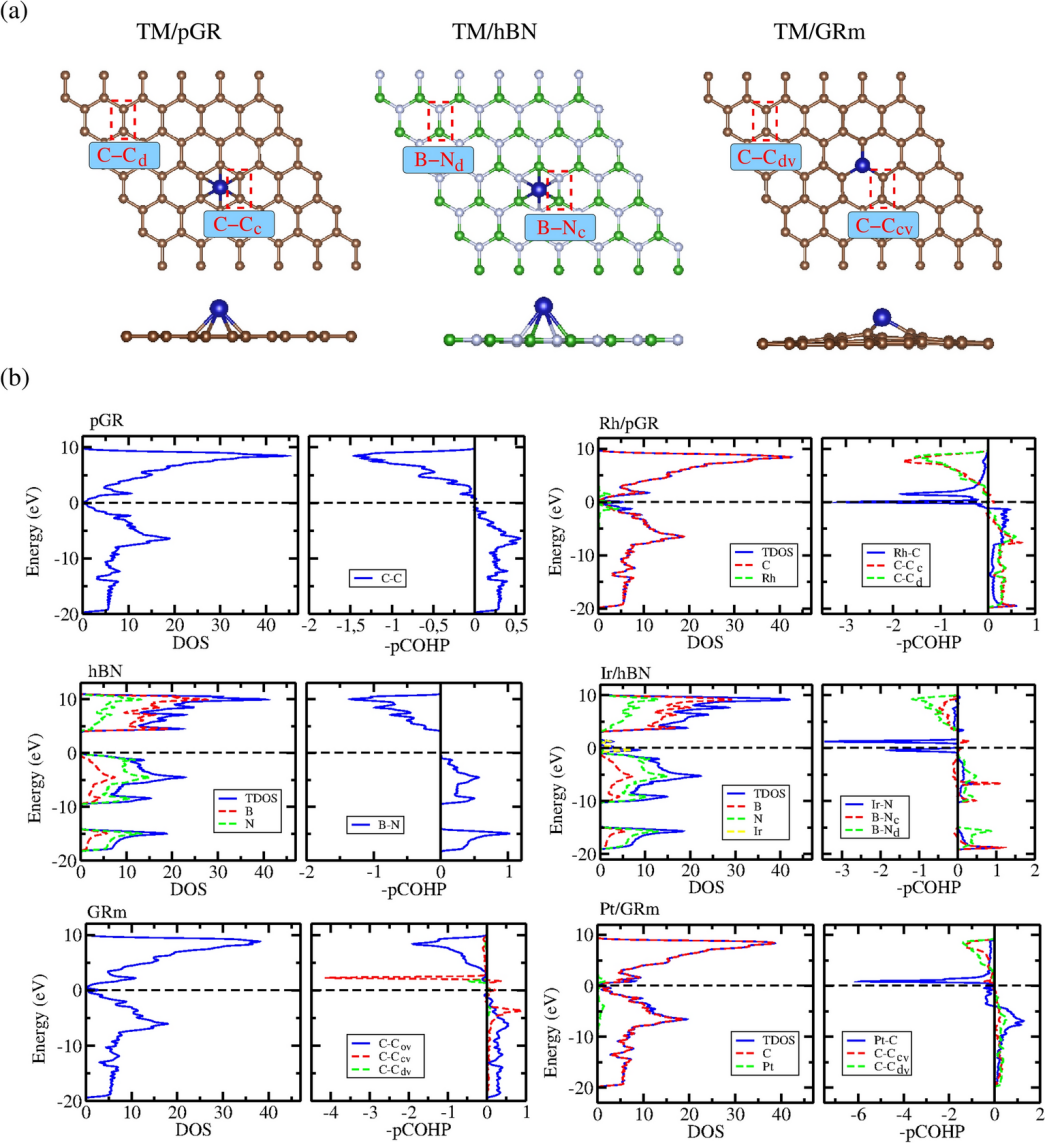
(**a**) Schematic representation of the (substrate atom)-(substrate atom) chemical bonds in regions distant (C-$\text {C}_\text {d}$ and B-$\text {N}_\text {d}$) and close (C-$\text {C}_\text {c}$ and B-$\text {N}_\text {c}$) to the TM. (**b**) DOS and -pCOHP plots for pGR, Rh/pGR, hBN, Ir/hBN, GRm, and Pt/GRm (complete cases are shown in the Supporting Information , Figures S5 − S7). For GRm, the analysis includes C-C bonds outside the vacancy (C-$\text {C}_\text {ov}$), distant (C-$\text {C}_\text {dv}$), and close (C-$\text {C}_\text {cv}$).

To explore the interaction mechanisms, electronic modifications, and bonding characteristics of TM adsorption, we analyzed the DOS and -pCOHP of these systems. Figure 4 illustrates the electronic properties of pristine substrates and representative TM/ sub-systems with the highest adsorption energy magnitudes. The Supporting Information (Figures S5−S7) display the analyses for all lowest energy configurations. The DOS analysis includes total DOS (TDOS) and local DOS contributions from the substrate and TM atoms relative to the Fermi energy ($E_\text {F} = {0}\,eV$). The -pCOHP analysis focuses on TM–substrate and substrate–substrate bonding interactions, distinguishing distant (C-C$\text {d}$ and B-N$\text {d}$) and close (C-C$\text {c}$ and B-N$\text {c}$) bonds, as defined in Figure 4(a). In Figure 4(b), we present the DOS and -pCOHP plots for pGR, Rh/pGR, hBN, Ir/hBN, GRm, and Pt/GRm.

The DOS for pristine pGR shows a steep decline close to $E_\text {F}$, in line with its semimetallic nature and distinctive Dirac cone (V-shaped behavior). The absence of a bandgap is confirmed by prominent peaks in the valence and conduction bands, respectively, at about -7 eV and 9 eV. TM adsorption, as seen for Rh/pGR, introduces new electronic states near $E_\text {F}$ due to hybridization between TM *d*-orbitals and graphene’s $\pi$-system, enhancing conductivity while preserving the semimetallic character.

Pristine hBN, in contrast, has a wide bandgap of approximately 4.7 eV, reflecting its insulating nature. N orbitals are the main source of DOS contributions below $E_\text {F}$, whereas B orbitals predominate above it. Localized states are introduced close to $E_\text {F}$ by TM adsorption, as in Ir/hBN, which modifies the bandgap slightly while preserving the overall insulating nature. In contrast to graphene-based substrates, the interactions involving N lone pairs and unoccupied B orbitals result in poorer adsorption.

Because of its monovacancy, the GRm substrate shows notable electronic changes, introducing new states close to $E_\text {F}$ and moving toward metallic behavior. TM adsorption, as demonstrated by Pt/GRm, intensifies this effect by exhibiting a high hybridization between the vacancy-modified graphene and TM *d*-orbitals. This leads to strong covalent bonds and improved electrical conductivity, which results in GRm being a good option for catalytic applications.

The -pCOHP analysis provides insights into bonding and anti-bonding interactions. For TM/pGR, bonding states lie below $E_\text {F}$ and anti-bonding states above it, indicating moderate covalent character in TM–C bonds, as shown for Rh/pGR. TM adsorption induces localized strain, weakening C-C$_\text {c}$ bonds more than C-C$_\text {d}$ bonds, yet the TM–C interaction remains stable due to substantial orbital overlap.

For TM/hBN, -pCOHP analysis reveals predominantly bonding states below $E_\text {F}$, with slight anti-bonding contributions near the adsorption site. TM–N and TM–B interactions exhibit mixed bonding and anti-bonding states, reinforcing the weaker TM interaction with hBN compared to graphene-based substrates.

The GRm case shows the strongest TM–substrate interactions, as evidenced by pronounced bonding states below $E_\text {F}$ and corresponding anti-bonding states above it. The C-C bonds near the vacancy (C-$\text {C}_\text {cv}$) display increased anti-bonding character, reflecting significant lattice distortion that weakens local C-C bonds while enhancing adsorption-site reactivity.

As a result, TM adsorption-induced substrate-specific electronic changes are revealed by DOS and -pCOHP studies. The most noticeable alterations occur in GRm, which transforms to metallic behavior due to lattice distortion and strong TM–C contacts. On the other hand, with only minor alterations in hBN and moderate alterations in pGR, both pGR and hBN maintain a large portion of their original electrical characteristics. The electronic architectures of pGR and hBN give rise to their different adsorption tendencies, with hBN’s N lone pairs and graphene’s delocalized $\pi$-system playing essential roles in TM binding. These results emphasize how crucial substrate modification and selection are for adjusting electronic characteristics in catalytic and associated applications.

#### Charge and hybridization analysis

**Fig. 5:**
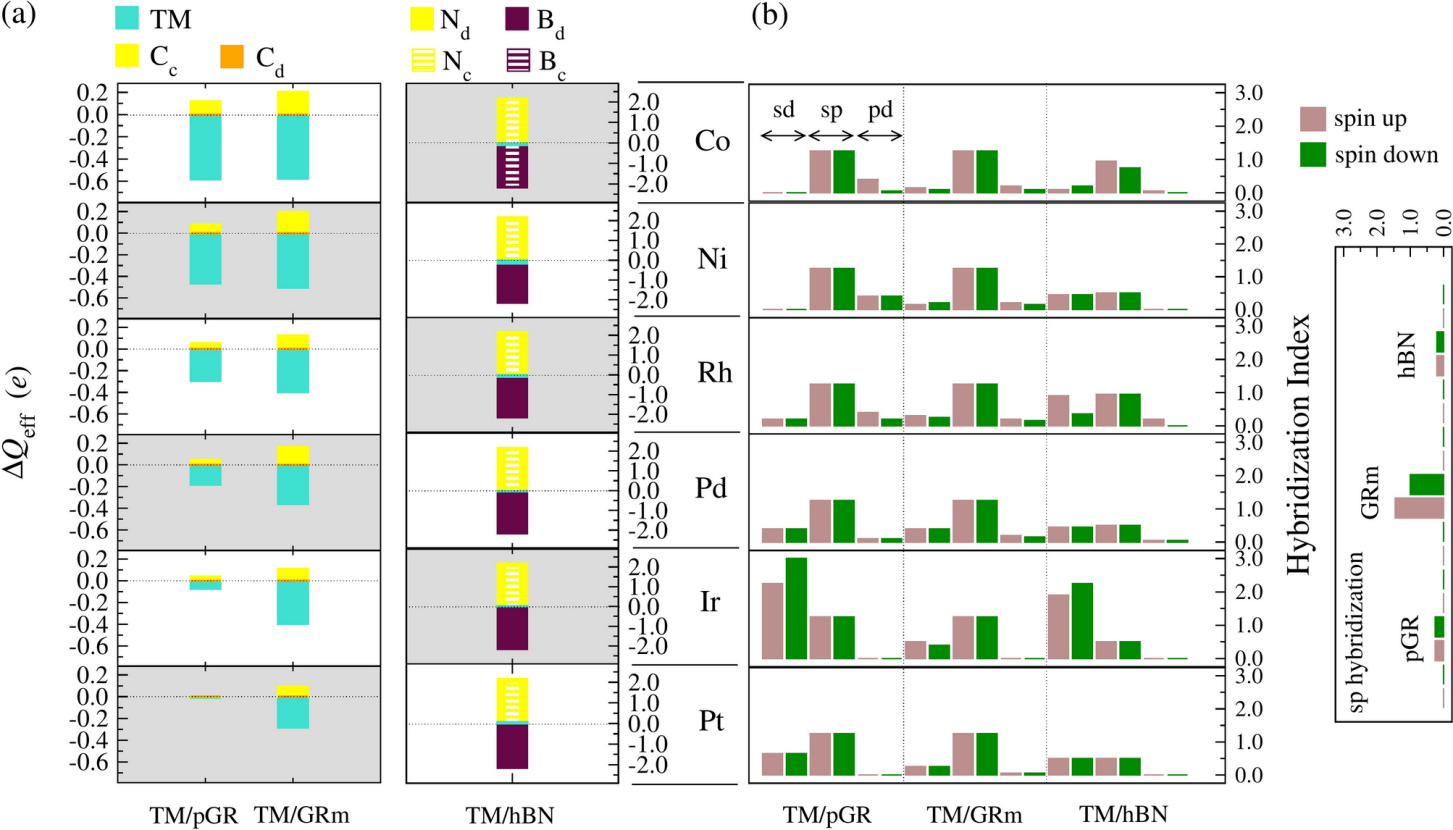
(**a**) Bader charge analysis ($\Delta Q_{\text {eff}}$) and (**b**) hybridization index (*hyb*) for the lowest energy TM/sub systems, where TM = Co, Ni, Rh, Pd, Ir, and Pt; and sub = pGR, hBN, and GRm.

To further explore the electronic characteristics of the systems, Figure 5(a) presents representative Bader charge analysis ($\Delta Q_{\text {eff}}$) for the TM/sub-systems, with detailed charge distributions provided in the Supporting Information  (Table S11, considering the average charge values of both substrate-atoms: directly involved and not involved in the interaction with the TM adatoms). These values quantify charge redistribution upon TM adsorption, where higher $\Delta Q_{\text {eff}}$ magnitudes indicate stronger electrostatic TM–substrate interactions and significant electron transfer, modifying the electronic properties of the substrate.

As seen in the DOS and -pCOHP investigations, their near-zero charge values confirm the stability of pristine substrates. Interacting C atoms in TM/pGR systems often have positive charges, whereas non-interacting atoms have no charge. Because graphene has a stronger electronegativity than other materials, charge transfer from the substrate causes all TMs to display negative charges. The charge transfer (in magnitude) decreases with increasing TM atomic number, consistent with reduced orbital overlap for larger TMs.

The $\Delta Q_{\text {eff}}$ values for TM/GRm systems are greater than those for TM/pGR, indicating stronger TM–substrate interactions made possible by the monovacancy structure. TMs are still negatively charged, but the C atoms close to the vacancy are positively charged. Localized electronic states at the vacancy interact significantly with TMs and are responsible for this enhanced charge transfer.

In contrast, TM/hBN systems exhibit minimal charge transfer, consistent with hBN’s insulating nature. For Co/hBN, where the TM adsorbs at the hollow site, B atoms carry negative charges and N atoms positive, leading to a cationic charge on Co. In systems where TMs adsorb at the N top site, the interacting N atom is positively charged. The charges of TMs vary, following: Ir and Pt have positive charges, while Co, Ni, Rh, and Pd have negative charges. This aligns with the -pCOHP study, which indicates that the anti-bonding contributions of Ir and Pt are more significant. This behavior is controlled by electronegativity differences between N, B, and TMs. In the case of the GRm, it facilitates the highest charge transfer across all TMs, emphasizing the role of vacancies in enhancing adsorption. While pGR supports moderate charge transfer, GRm’s modified structure enables stronger interactions. On the other hand, weak interactions on hBN maintain its stability by causing minimal charge transfer.

The most stable TM/sub systems’ *sd*, *sp*, and *pd* hybridization indices (considering spin-up and spin-down components) are shown in Figure 5(b), which also provides insights on their magnetic characteristics by exposing orbital mixing among *s*, *p*, and *d* orbitals. Pristine substrates exhibit *sp* hybridization, consistent with their $sp^2$-hybridized structures. Vacancies in GRm increase hybridization indices and break spin symmetry, enhancing reactivity while reducing the local stability.

In TM/pGR systems, *sp* and *pd* hybridizations dominate, indicating strong interactions between TM *d*-orbitals and graphene’s $\pi$-system. The *sd* hybridization is moderate but increases with heavier TMs. The *pd* hybridization is significant for Co, Ni, and Rh but decreases for Pd, Ir, and Pt, where *sd* hybridization becomes more prominent due to greater shielding effects, reducing orbital overlap with graphene.

In GRm systems, *pd* hybridization is enhanced, particularly in the spin-up channel, reflecting stronger TM–C interactions facilitated by the monovacancy. The *sp* hybridization becomes more symmetric between spin-up and spin-down components, indicating a stabilization effect as TMs integrate into the substrate.

For TM/hBN systems, *sp* hybridization dominates, with lower *pd* contributions than graphene-based substrates, indicating weaker interactions between TMs and hBN (between N *p*-orbitals and TM *d*-orbitals). Spin-dependent hybridization indices show minimal variation, except for Co/hBN, where spin asymmetry suggests lower stability. *sd* hybridization, which reflects localized TM–B/N interactions, is significant across all TMs.

The degree of *pd* hybridization indicates the strength of the TM–substrate interaction. The highest *pd* hybridization is found in GRm, indicating strong bonding necessary for catalytic applications. Moderate electronic changes are made possible by the balanced *sp* and *pd* hybridization in pGR, which maintains graphene’s semimetallic character while increasing reactivity. On the other hand, hBN’s lesser hybridization highlights how well-suited it is for uses that call for stable, barely disturbed substrates.

## Discussion and conclusions

We presented a comprehensive analysis of the TM single-atom adsorption on graphene-derived substrates (pGR, hBN, and GRm) using the DFT-PBE+D3 calculation protocol. We sought to integrate the intrinsic stability of these 2D substrates with the high reactivity of TMs (Co, Ni, Rh, Pd, Ir, and Pt), aiming to achieve the balance between stability and reactivity, which is crucial for catalytic applications. To this end, we investigated the adsorption properties, energy decomposition, electronic structure, charge transfer, and hybridization tendencies to uncover the underlying interaction mechanisms.

Through the adsorption energy analysis, we obtained that the interaction behaviors were substrate-dependent. In pGR and hBN, we observed the occurrence of weak chemisorption and physisorption, respectively, with $E_{\text {ads}}$ ranging from -1.05 eV to -1.80 eV for pGR and -0.51 eV to -1.74 eV for hBN, with both substrates preserving their electronic properties. On the other hand, GRm showed significantly stronger chemisorption, with $E_{\text {ads}}$ reaching up to -9.11 eV (Ir/GRm), a fact that occurs due to the monovacancy, which acts to increase the reactivity through defective states and more substantial orbital overlap.

From the electronic structure analyses (DOS and -pCOHP), we were able to demonstrate substrate-specific modifications in the adsorption of TMs. In pGR, the semimetallic nature is largely preserved, with moderate *pd* hybridization introducing states close to the Fermi level. In hBN, smaller bandgap reductions, but still with the maintenance of the insulating character and the presence of localized states, reflect weaker TM–substrate interactions. In the case of GRm, we found that it undergoes a transition to metallic behavior due to the strong TM–C interactions and vacancy-induced defect states close to the Fermi level, facilitating charge redistribution. -pCOHP analysis also confirmed the covalent TM–C bonds, with strong bonding states below and antibonding states above the Fermi level.

We determined the charge transfer patterns with Bader charge calculations, which demonstrated that pGR and GRm promote charge flow from the substrate to the TMs. Because of its reactive defect structure, GRm shows the maximum charge transfer. On the other hand, charge transfer in hBN was negligible, which is in line with its inert and insulating nature. According to hybridization index calculations, there is evidence of improved *pd* hybridization in GRm, moderate hybridization in pGR, and weaker *pd* contributions in exchange for higher *sp* hybridization in hBN.

GRm emerged as the most promising among the SACs substrate due to its strong anchoring for transition metals (TMs), efficient charge transfer, and metallic electronic states. The presence of monovacancies in GRm enhances TM capture, preventing aggregation and increasing overall reactivity. In contrast, pGR showed moderate stability and reactivity, while hBN remained inert, suitable for dielectric applications. Late TMs like Pt and Rh demonstrated significant interactions and contributions from *d*-orbitals across all substrates. Based on that, the importance of defect engineering is evident, particularly the role of monovacancies in graphene in enhancing TM binding strength and reactivity, contributing to the design of tailored catalytic materials.

Our findings reveal that the SAC reactivity is governed primarily by substrate-dependent adsorption strength, charge transfer, and electronic structure alteration. GRm with monovacancy defect possesses the highest adsorption energy magnitudes, favoring robust TM–C bonds, metallic-like behavior with augmented charge redistribution, and catalytic activity. Bader charge analysis confirms that GRm is an adequate electron reservoir, while negligible charge transfer limits its reactivity in hBN. DOS and pCOHP computations also reveal that strong *pd* hybridization in GRm enhances covalent TM–C bonding to maximize metal–support interaction in catalysis and moderate hybridization in pGR to provide equilibrium between stability and reactivity. Our stability computations also reveal that adsorption energies in pGR and hBN are smaller than both TM–TM dimer binding and bulk cohesive energies, implying enhanced aggregation tendency. Conversely, in GRm, adsorption energy is larger than dimer and bulk energies. This confirms that defect engineering through monovacancies in graphene and stabilizing SACs by preventing clustering enhances reactivity through enhanced metal–support interactions. Late TMs such as Pt and Rh, with significant *d*-orbital contributions, possess the strongest adsorption and electronic interactions on all substrates, advocating for their catalytic potential as active sites. These findings identify that reactivity is not merely determined by the inherent character of the TM but also by support since defected graphene yields optimal balance for catalytic application through stable yet reactive SACs.

## Supplementary Information

Supplementary Information.

## Data Availability

The data supporting this study’s findings are available within the article and its supplementary materials. Additional datasets used during the current study are available upon reasonable request. If you wish to request further data or clarifications, please get in touch with Dr. Celso Ricardo Caldeira Rêgo (celso.rego@kit.edu) or Dr. Maurício J. Piotrowski (mauriciomjp@gmail.com).
