# Supplementary material for: Understanding stability and reactivity of transition metal single-atoms on graphene
Source: Sci Rep. 2025 May 3;15:15496. doi: 10.1038/s41598-025-00126-y (PMC12049553; doi:10.1038/s41598-025-00126-y)
Supplement: Supplementary file 1 — Supplementary Information. [file 41598_2025_126_MOESM1_ESM.pdf]

Supporting Information: Understanding Stability and Reactivity of Transition Metal Single-Atoms on Graphene

Wesley Oliveira Morais,† João Paulo Cerqueira Felix,‡ Gabriel Reynald da Silva,¶ Carlos Maciel de Oliveira Bastos,§ Alexandre C. Dias,§ Efracio Mamani Flores,‖ Celso R. C. Rêgo,*,⊥ Vinícius da Silva Ramos de Sousa,‡ Diego Guedes-Sobrinho,¶ and Maurício J. Piotrowski†

†*Department of Physics, Federal University of Pelotas, PO Box* 354, 96010-900, *Pelotas, RS, Brazil*

‡*Institute of Physics Armando Dias Tavares, Rio de Janeiro State University,* 20550-900, *Rio de Janeiro, RJ, Brazil*

¶*Chemistry Department, Federal University of Paraná,* 81531 − 980, *Curitiba, PR, Brazil*

§*Institute of Physics and International Center of Physics, University of Brasília,* 70919 − 970, *Brasília, DF, Brazil*

‖*Department of Physics, Jorge Basadre Grohmann National University, Tacna, Peru*

⊥*Institute of Nanotechnology Hermann-von-Helmholtz-Platz, Karlsruhe Institute of Technology,* 76021, *Karlsruhe, Germany*

E-mail: celso.rego@kit.edu

Contents

1 Convergence Tests **S3**

2 Combined Systems - Adsorption Properties **S6**

3 Combined Systems - In-depth Energy Analysis **S13**

4 Combined Systems - Electronic Analysis **S14**

5 Combined Systems - Bader Charge Analysis **S17**

1 Convergence Tests

Below, we present the convergence tests conducted for the unit cell, supercell size, **k**-mesh, number of plane waves (ENCUT), and energy (EDIFF) and atomic force (EDIFFG) criteria. The first test involved a comparison of our unit cell (Table S1), simulated using DFT-PBE+D3 calculations. Our results align well with those from reference studies.

Table S1: Test for the graphene unit cell: number of carbon atoms ($\mathbf{C}_n$), lattice parameters (**a**, **b** and **c**), angles (α, β and γ), volume (**V**), C-C distance ($\mathbf{d}_{\text{C-C}}$), and binding energy $\mathbf{E}_\text{b}$.

**Graphene unit cell (this work):**							
$\mathbf{C}_n$	$\mathbf{a} = \mathbf{b}$ (Å)	**c** (Å)	$\alpha = \beta$ (°)	γ (°)	**V** (Å^3)	$\mathbf{d}_{\text{C-C}}$ (Å)	$\mathbf{E}_\text{b}$ (eV)
2	2.467	16.997	90	120	89.590	1.424	-7.844
**Graphene unit cell (reference works):**							
$\mathbf{C}_n$	$\mathbf{a} = \mathbf{b}$ (Å)	**c** (Å)	$\alpha = \beta$ (°)	γ (°)	**V** (Å^3)	$\mathbf{d}_{\text{C-C}}$ (Å)	$\mathbf{E}_\text{b}$ (eV)
2	2.461	17.000	90	120	89.167	1.420	-7.878

For the second test, we aimed to determine the distances between two Co atoms in a supercell (Table S2). The interaction between these replicated atoms in the supercell should be minimized.

Table S2: Test for the supercell using **k**-mesh $4 \times 4 \times 1$ and cutoff energy of 600 eV. Parameters include the number of carbon atoms ($\mathbf{C}_n$), supercell size (**size**), lattice parameters (**a**, **b** and **c**), angles (α, β and γ) and the distance between two Co atoms ($\mathbf{d}_{\text{Co-Co}}$).

$\mathbf{C}_n$	**size**	$\mathbf{a} = \mathbf{b}$ (Å)	**c** (Å)	$\alpha = \beta$ (°)	γ (°)	$\mathbf{d}_{\text{Co-Co}}$
18	$3 \times 3 \times 1$	7.401	17.000	90	120	7.401
32	$4 \times 4 \times 1$	9.868	17.000	90	120	9.868
50	$5 \times 5 \times 1$	12.335	17.000	90	120	12.335
72	$6 \times 6 \times 1$	14.802	17.000	90	120	14.802
98	$7 \times 7 \times 1$	17.269	17.000	90	120	17.269
128	$8 \times 8 \times 1$	19.736	17.000	90	120	19.736

In the third and fourth tests, we aimed to determine the convergence of physical quantities (binding energy, $\mathbf{E}_\text{b}$; adsorption energy, $\mathbf{E}_\text{ads}$; and total magnetic moment, $\mathbf{m}_\text{T}$) using the **k**-point mesh based on the Monkhorst-Pack technique (Table S3 and Table S4). The fifth test focused on determining the convergence of physical properties with the number of plane waves in the supercell (Table S5). Bloch functions can be expanded into an infinite sum of plane waves in reciprocal space. In practice, only Bloch functions with kinetic energies below a cutoff value (ENCUT) are considered.

The sixth test conducted was the energy convergence test, EDIFF (Table S6). EDIFF

Table S3: Test of **k**-mesh with pristine graphene (pGR) and Co/pGR for the $4 \times 4 \times 1$ supercell and a cutoff energy of 600 eV: binding energy ($\mathbf{E}_b$), adsorption energy ($\mathbf{E}_{ads}$), and total magnetic moment ($\mathbf{m}_T$).

C_{32}	**pGR**		**Co/pGR**	
**k**-mesh	$\mathbf{E}_b$ (eV)	$\mathbf{m}_T$ (μ_B)	$\mathbf{E}_{ads}$ (eV)	$\mathbf{m}_T$ (μ_B)
$1 \times 1 \times 1$	-7.894	0.000	-0.874	1.000
$2 \times 2 \times 1$	-7.879	0.000	-1.099	1.000
$3 \times 3 \times 1$	-7.875	0.444	-1.209	1.444
$4 \times 4 \times 1$	-7.878	0.000	-1.115	1.000
$5 \times 5 \times 1$	-7.878	0.000	-1.110	1.000
$6 \times 6 \times 1$	-7.878	0.000	-1.111	1.000
$7 \times 7 \times 1$	-7.878	0.000	-1.111	1.000
$\mathbf{a} = \mathbf{b}$ (Å)	**c** (Å)	$\alpha = \beta$ (°)	γ (°)	**V** (Å^3)
9.868	17.000	90	120	1433.689

Table S4: Test of **k**-mesh with pGR and Co/pGR for the $5 \times 5 \times 1$ supercell and a cutoff energy of 600 eV: binding energy ($\mathbf{E}_b$), adsorption energy ($\mathbf{E}_{ads}$), and total magnetic moment ($\mathbf{m}_T$).

C_{50}	**pGR**		**Co/pGR**	
**k**-mesh	$\mathbf{E}_b$ (eV)	$\mathbf{m}_T$ (μ_B)	$\mathbf{E}_{ads}$ (eV)	$\mathbf{m}_T$ (μ_B)
$1 \times 1 \times 1$	-7.866	0.000	-1.092	1.000
$2 \times 2 \times 1$	-7.877	0.000	-1.152	1.000
$3 \times 3 \times 1$	-7.876	0.444	-1.218	1.444
$4 \times 4 \times 1$	-7.878	0.000	-1.136	1.000
$5 \times 5 \times 1$	-7.878	0.000	-1.139	1.000
$6 \times 6 \times 1$	-7.878	0.000	-1.135	1.000
$7 \times 7 \times 1$	-7.878	0.000	-1.116	1.001
$\mathbf{a} = \mathbf{b}$ (Å)	**c** (Å)	$\alpha = \beta$ (°)	γ (°)	**V** (Å^3)
12.335	17.000	90	120	2240.138

Table S5: Cutoff energy test for the plane wave basis (ENCUT) with pGR and Co/pGR using a **k**-mesh of $4 \times 4 \times 1$: binding energy ($\mathbf{E}_b$), adsorption energy ($\mathbf{E}_{ads}$), and total magnetic moment ($\mathbf{m}_T$).

C_{32}	**pGR**		**Co/pGR**	
**ENCUT** (eV)	$\mathbf{E}_b$ (eV)	$\mathbf{m}_T$ (μ_B)	$\mathbf{E}_{ads}$ (eV)	$\mathbf{m}_T$ (μ_B)
250	-7.860	0.000	-0.788	1.000
300	-7.919	0.000	-1.082	1.000
350	-7.897	0.444	-1.107	1.000
400	-7.884	0.000	-1.113	1.000
450	-7.878	0.000	-1.115	1.000
500	-7.875	0.000	-1.132	1.000
550	-7.876	0.000	-1.108	1.000
600	-7.878	0.000	-1.120	1.000
650	-7.880	0.000	-1.121	1.000
700	-7.882	0.000	-1.124	1.000
$\mathbf{a} = \mathbf{b}$ (Å)	**c** (Å)	$\alpha = \beta$ (°)	γ (°)	**V** (Å^3)
9.868	17.000	90	120	1433.689

represents the stopping condition for the self-consistent electronic loop or, alternatively, the relaxation of the electronic degrees of freedom.

Table S6: Convergence test for the total energy difference (EDIFF) with pGR and Co/pGR using a **k**-mesh of $4 \times 4 \times 1$ and a cutoff energy of 600 eV: binding energy ($\mathbf{E}_b$), adsorption energy ($\mathbf{E}_{ads}$), and total magnetic moment ($\mathbf{m}_T$).

C_{32}	**pGR**		**Co/pGR**	
**EDIFF** (eV)	$\mathbf{E}_b$ (eV)	$\mathbf{m}_T$ (μ_B)	$\mathbf{E}_{ads}$ (eV)	$\mathbf{m}_T$ (μ_B)
10^{-2}	-7.878	0.005	-1.129	1.000
10^{-3}	-7.878	0.002	-1.115	1.000
10^{-4}	-7.878	0.000	-1.115	1.000
10^{-5}	-7.878	0.000	-1.115	1.000
10^{-6}	-7.878	0.000	-1.115	1.000
10^{-7}	-7.878	0.000	-1.115	1.000
10^{-8}	-7.878	0.000	-1.115	1.000
$\mathbf{a} = \mathbf{b}$ (Å)	$\mathbf{c}$ (Å)	$\alpha = \beta$ (°)	γ (°)	$\mathbf{V}$ (Å^3)
9.868	17.000	90	120	1433.689

The seventh and final test conducted was the convergence of atomic forces, EDIFFG (Table S7). EDIFFG represents the stopping condition for the ionic self-consistent loop or, alternatively, the relaxation of the structural degrees of freedom, minimizing the force between atoms in the structure.

Table S7: Convergence test for atomic forces (EDIFFG) with pGR and Co/pGR using a **k**-mesh of $4 \times 4 \times 1$ and a cutoff energy of 600 eV: binding energy ($\mathbf{E}_b$), adsorption energy ($\mathbf{E}_{ads}$), and total magnetic moment ($\mathbf{m}_T$).

C_{32}	**pGR**		**Co/pGR**	
**EDIFFG** (eV/Å)	$\mathbf{E}_b$ (eV)	$\mathbf{m}_T$ (μ_B)	$\mathbf{E}_{ads}$ (eV)	$\mathbf{m}_T$ (μ_B)
-0.050	-7.878	0.000	-1.115	1.001
-0.035	-7.878	0.000	-1.115	1.001
-0.025	-7.878	0.000	-1.115	1.001
-0.020	-7.878	0.000	-1.115	1.001
-0.015	-7.878	0.000	-1.115	1.000
-0.010	-7.878	0.000	-1.115	1.000
-0.005	-7.878	0.000	-1.115	1.000
$\mathbf{a} = \mathbf{b}$ (Å)	$\mathbf{c}$ (Å)	$\alpha = \beta$ (°)	γ (°)	$\mathbf{V}$ (Å^3)
9.868	17.000	90	120	1433.689

2 Combined Systems - Adsorption Properties

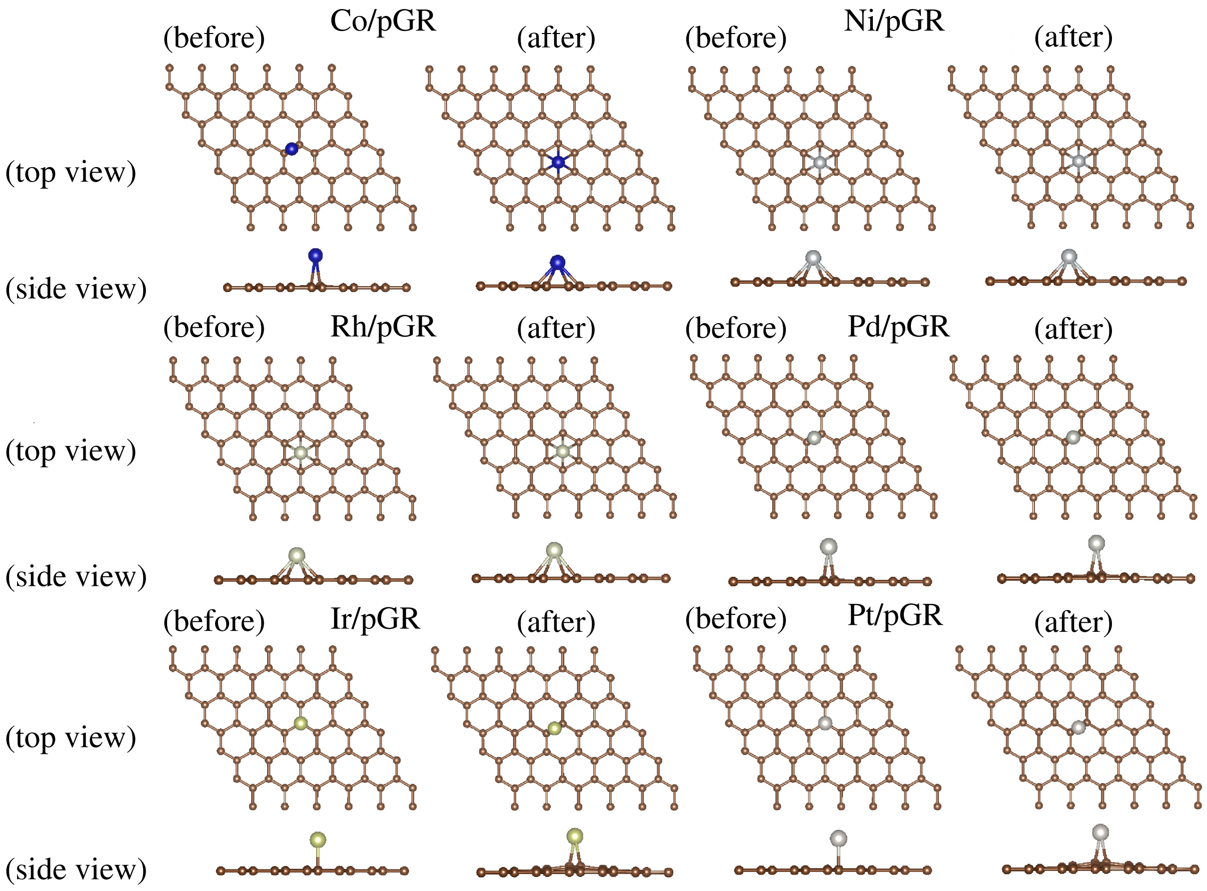

Figure S1: The most stable configurations (before and after optimization) of the adsorption of transition metals (TMs) on the pGR substrate.

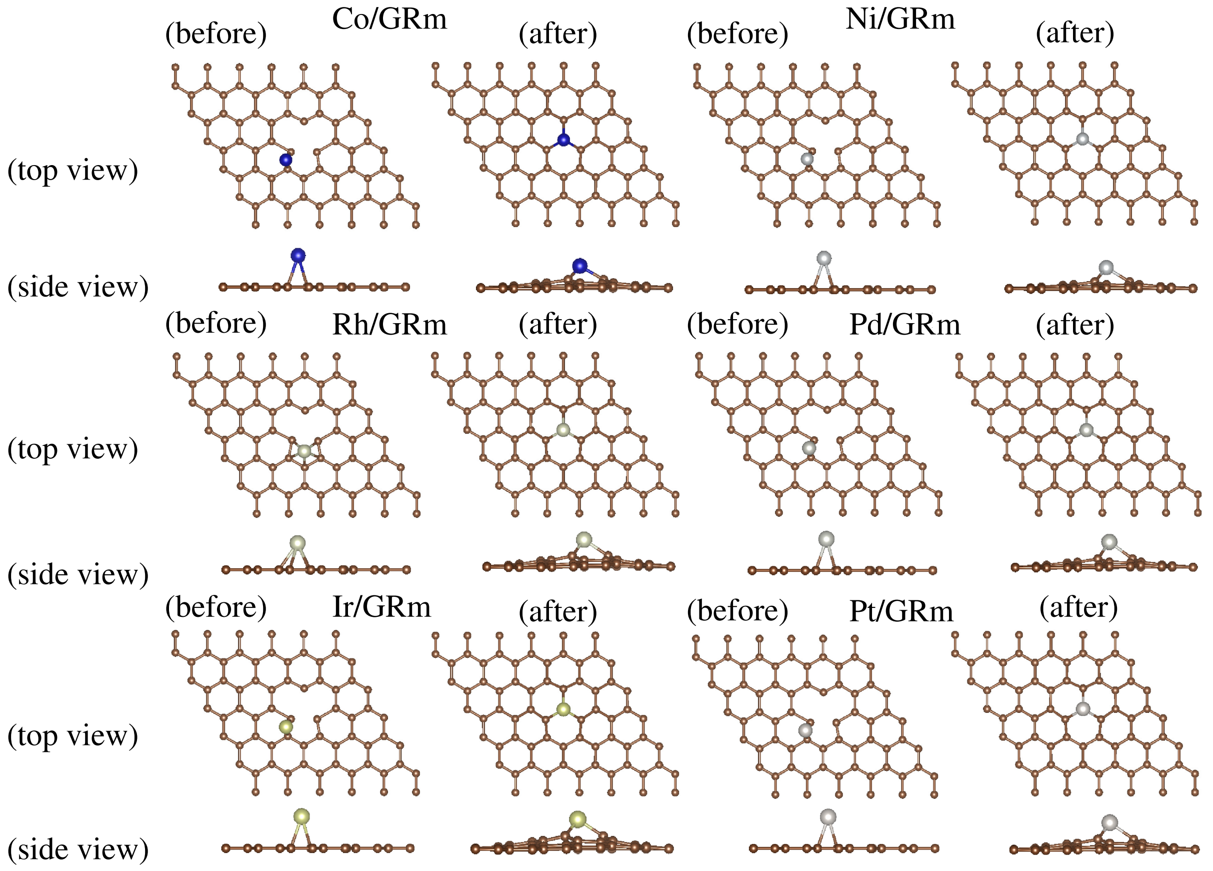

Figure S2: The most stable configurations (before and after optimization) of the adsorption of transition metals (TMs) on the GRm substrate.

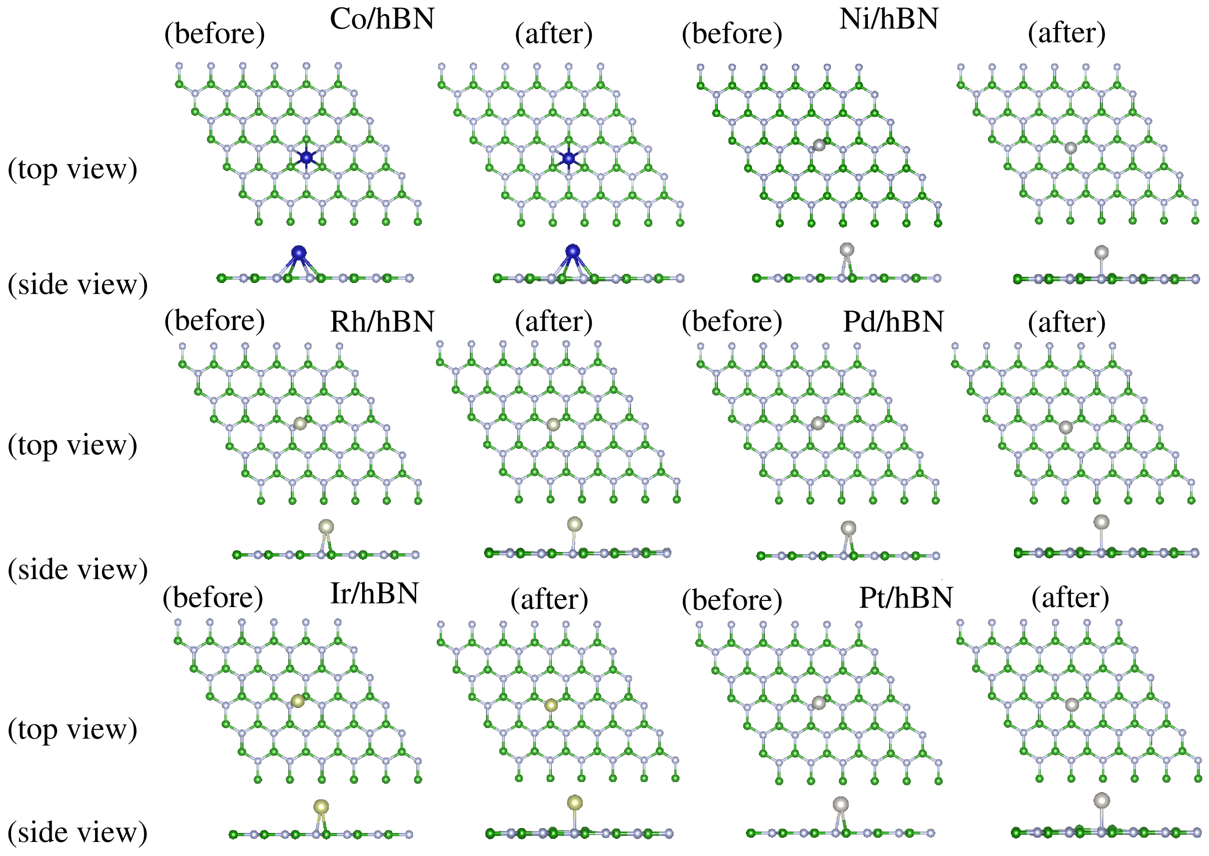

Figure S3: The most stable configurations (before and after optimization) of the adsorption of transition metals (TMs) on the hBN substrate.

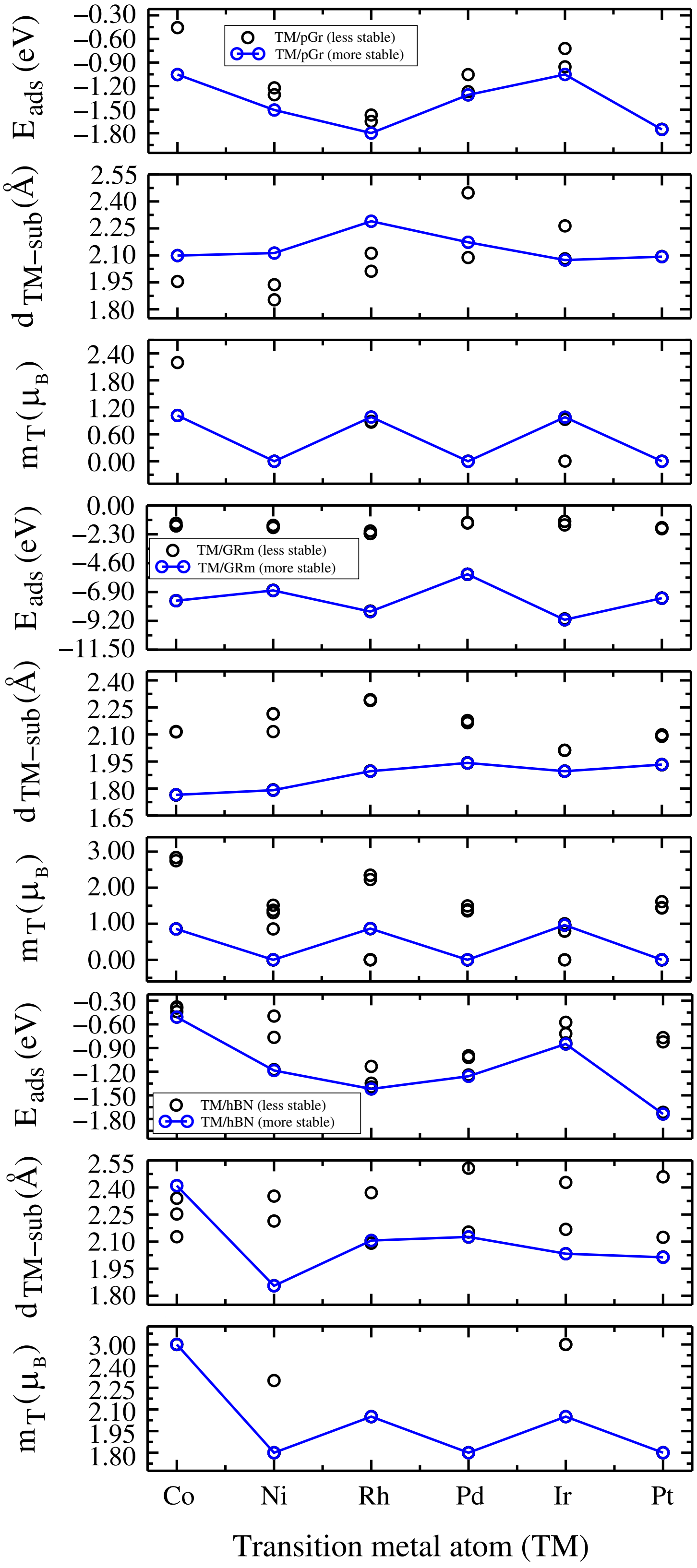

Figure S4: Adsorption energy, E_{ads}, distance between the TM atom and the substrate, $d_{\text{TM-sub}}$, and total magnetic moment, m_T, for adsorption on pGR, GRm, and hBN, respectively. The blue lines represent the values for the most stable TM/sub systems, while the black circles indicate the less stable configurations.

Table S8: Properties of TM adsorption on pGR and hBN: total relative energy (ΔE_T), adsorption energy (E_{ads}), total magnetic moment (m_T), and distance between the TM and the substrate (d_{TM}).

System	site	ΔE_T (eV)	E_{ads} (eV)	m_T (μ_B)	d_{TM} (Å)
Co/pGR	top	0.596	-0.456	2.197	1.955
	bridge → hollow	0.000	-1.053	1.019	2.099
	hollow	0.000	-1.053	1.020	2.099
Ni/pGR	top	0.282	-1.222	0.000	1.853
	bridge	0.194	-1.310	0.000	1.937
	hollow	0.000	-1.504	0.000	2.113
Rh/pGR	top	0.230	-1.568	0.889	2.012
	bridge	0.149	-1.649	0.870	2.112
	hollow	0.000	-1.798	0.984	2.290
Pd/pGR	top	0.042	-1.271	0.000	2.088
	bridge	0.000	-1.312	0.000	2.173
	hollow	0.258	-1.054	0.000	2.448
Ir/pGR	top → bridge	0.000	-1.052	0.981	2.074
	bridge	0.097	-0.955	0.932	2.081
	hollow	0.331	-0.722	0.004	2.264
Pt/pGR	top → bridge	0.000	-1.752	0.000	2.093
	bridge	0.002	-1.750	0.000	2.094
	hollow → bridge	0.002	-1.750	0.000	2.094
Co/hBN	top^B	0.128	-0.379	3.000	2.127
	top^N	0.068	-0.439	3.000	2.340
	bridge	0.114	-0.393	3.000	2.253
	hollow	0.000	-0.508	3.000	2.410
Ni/hBN	top^B	0.690	-0.495	2.000	2.352
	top^N	0.012	-1.174	0.000	1.856
	bridge → top^N	0.000	-1.185	0.000	1.855
	hollow	0.421	-0.765	0.000	2.214
Rh/hBN	top^B	0.286	-1.133	1.000	2.091
	top^N	0.025	-1.394	1.000	2.106
	bridge → top^N	0.000	-1.419	1.000	2.106
	hollow	0.073	-1.346	1.000	2.371
Pd/hBN	top^B	0.238	-1.020	0.000	2.153
	top^N	0.017	-1.241	0.000	2.125
	bridge → top^N	0.000	-1.258	0.000	2.126
	hollow	0.260	-0.998	0.000	2.507
Ir/hBN	top^B	0.276	-0.574	3.000	2.168
	top^N	0.014	-0.835	1.000	2.033
	bridge → top^N	0.000	-0.849	1.000	2.033
	hollow	0.131	-0.718	3.000	2.428
Pt/hBN	top^B	0.970	-0.768	0.000	2.124
	top^N	0.021	-1.715	0.000	2.015
	bridge → top^N	0.000	-1.737	0.000	2.013
	hollow	0.915	-0.822	0.000	2.459

Table S9: Properties of TM adsorption on GRm: total relative energy (ΔE_T), adsorption energy (E_{ads}), total magnetic moment (m_T), and distance between the TM and the substrate (d_{TM}).

System	site	ΔE_T (eV)	E_{ads} (eV)	m_T (μ_B)	d_{TM} (Å)
Co/GRm	top FMV → hollow	5.965	-1.628	2.834	2.116
	top MV1 → embedded	0.002	-7.591	0.856	1.765
	top MV2 → embedded	0.002	-7.592	0.856	1.765
	bridge FMV → hollow	5.963	-1.630	2.838	2.116
	bridge MV1 → embedded	0.000	-7.593	0.857	1.765
	bridge MV2 → embedded	0.001	-7.593	0.856	1.765
	hollow FMV	6.177	-1.416	2.747	2.114
	hollow MV1 → embedded	0.001	-7.592	0.857	1.765
	hollow MV2 → embedded	0.002	-7.591	0.857	1.764
	2CMV1 → embedded	0.004	-7.590	0.857	1.764
	2CMV2 → embedded	0.004	-7.590	0.856	1.765
	embedded	0.004	-7.590	0.856	1.765
Ni/GRm	top FMV → hollow	4.102	-1.582	1.376	2.215
	top MV1 → embedded	0.002	-6.771	0.856	1.792
	top MV2 → embedded	0.001	-6.771	0.000	1.792
	bridge FMV → hollow	4.123	-1.752	1.513	2.214
	bridge MV1 → embedded	0.000	-6.773	0.000	1.791
	bridge MV2 → embedded	0.001	-6.772	0.000	1.792
	hollow FMV	4.092	-1.650	1.306	2.116
	hollow MV1 → embedded	0.001	-6.772	0.000	1.791
	hollow MV2 → embedded	0.002	-6.771	0.000	1.792
	2CMV1 → embedded	0.003	-6.769	0.000	1.792
	2CMV2 → embedded	0.003	-6.769	0.000	1.792
	embedded	0.003	-6.769	0.000	1.792
Rh/GRm	top FMV → hollow	6.218	-2.240	2.345	2.290
	top MV1 → embedded	0.031	-8.427	0.000	1.897
	top MV2 → embedded	0.031	-8.428	0.002	1.897
	bridge FMV → hollow	6.220	-2.238	2.225	2.288
	bridge MV1 → embedded	0.030	-8.428	0.001	1.896
	bridge MV2 → embedded	0.030	-8.428	0.002	1.896
	hollow FMV	6.424	-2.034	2.346	2.293
	hollow MV1 → embedded	0.000	-8.458	0.866	1.896
	hollow MV2 → embedded	0.000	-8.458	0.865	1.896
	2CMV1 → embedded	0.001	-8.457	0.863	1.897
	2CMV2 → embedded	0.033	-8.425	0.000	1.896
	embedded	0.002	-8.456	0.866	1.896
Pd/GRm	top FMV → bridge	4.102	-1.383	1.388	2.171
	top MV1 → embedded	0.002	-5.484	0.000	1.942
	top MV2 → embedded	0.001	-5.484	0.000	1.942
	bridge FMV	4.123	-1.362	1.495	2.177
	bridge MV1 → embedded	0.000	-5.485	0.000	1.942
	bridge MV2 → embedded	0.001	-5.485	0.000	1.942
	hollow FMV → bridge	4.092	-1.394	1.356	2.165
	hollow MV1 → embedded	0.001	-5.485	0.000	1.942
	hollow MV2 → embedded	0.002	-5.484	0.000	1.942
	2CMV1 → embedded	0.003	-5.483	0.000	1.942
	2CMV2 → embedded	0.003	-5.483	0.000	1.942
	embedded	0.003	-5.482	0.000	1.942

Ir/GRm	top FMV	7.844	-1.266	0.798	2.012
	top MV1 → embedded	0.001	-9.110	0.964	1.896
	top MV2 → embedded	0.064	-9.047	0.000	1.896
	bridge FMV → top	7.559	-1.551	1.001	2.012
	bridge MV1 → embedded	0.000	- 9.111	0.965	1.896
	bridge MV2 → embedded	0.063	-9.047	0.000	1.896
	hollow FMV → top	7.845	-1.266	0.794	2.012
	hollow MV1 → embedded	0.000	-9.110	0.964	1.896
	hollow MV2 → embedded	0.001	-9.110	0.965	1.896
	2CMV1 → embedded	0.002	-9.109	0.964	1.896
	2CMV2 → embedded	0.001	-9.109	0.964	1.897
	embedded	0.003	-9.108	0.966	1.896
Pt/GRm	top FMV → bridge	5.566	-1.823	1.448	2.092
	top MV1 → embedded	0.001	-7.388	0.000	1.933
	top MV2 → embedded	0.001	-7.388	0.000	1.933
	bridge FMV	5.622	-1.767	1.613	2.098
	bridge MV1 → embedded	0.000	-7.389	0.000	1.933
	bridge MV2 → embedded	0.001	-7.388	0.000	1.933
	hollow FMV → bridge	5.534	-1.855	1.436	2.089
	hollow MV1 → embedded	0.001	-7.388	0.000	1.933
	hollow MV2 → embedded	0.001	-7.388	0.000	1.932
	2CMV1 → embedded	0.002	-7.387	0.000	1.933
	2CMV2 → embedded	0.001	-7.387	0.000	1.932
	embedded	0.002	-7.387	0.000	1.932

3 Combined Systems - In-depth Energy Analysis

Table S10: Properties of TM/(pGR, hBN, or GRm): interaction energies (ΔE_{int}), distortion energy (ΔE_{dis}), and adsorption energy (E_{ads}). Effective coordination number of the adsorbed substrates (ECN), change in ECN (ΔECN), average bond length of the adsorbed substrates (d_{av}), and change in d_{av} (Δd_{av}).

System	ΔE_{int} (eV)	ΔE_{dis} (eV)	E_{ads} (eV)	ECN	ΔECN (%)	d_{av} (Å)	Δd_{av} (%)
Co/pGR	-1.111	0.058	-1.053	3.000	0.000	1.424	0.000
Ni/pGR	-1.547	0.043	-1.504	3.000	0.000	1.424	0.000
Rh/pGR	-1.840	0.043	-1.797	3.000	0.000	1.424	0.000
Pd/pGR	-1.408	0.096	-1.312	3.000	0.000	1.425	-0.070
Ir/pGR	-1.350	0.298	-1.052	2.999	0.033	1.425	-0.070
Pt/pGR	-2.079	0.327	-1.752	2.999	0.033	1.425	-0.070
Co/GRm	-8.474	0.881	-7.593	2.957	-0.169	1.424	0.140
Ni/GRm	-7.447	0.675	-6.772	2.957	-0.169	1.423	0.210
Rh/GRm	-9.657	1.199	-8.458	2.957	-0.169	1.424	0.140
Pd/GRm	-6.342	0.857	-5.485	2.957	-0.169	1.423	0.210
Ir/GRm	-10.412	1.301	-9.111	2.957	-0.169	1.425	0.070
Pt/GRm	-8.472	1.083	-7.389	2.957	-0.169	1.424	0.140
Co/hBN	-0.526	0.018	-0.508	3.000	0.000	1.451	0.000
Ni/hBN	-1.330	0.145	-1.185	3.000	0.000	1.451	0.000
Rh/hBN	-1.581	0.162	-1.419	3.000	0.000	1.451	0.000
Pd/hBN	-1.319	0.061	-1.258	3.000	0.000	1.451	0.000
Ir/hBN	-1.063	0.214	-0.849	2.999	0.033	1.451	0.000
Pt/hBN	-1.953	0.216	-1.737	2.999	0.033	1.451	0.000

4 Combined Systems - Electronic Analysis

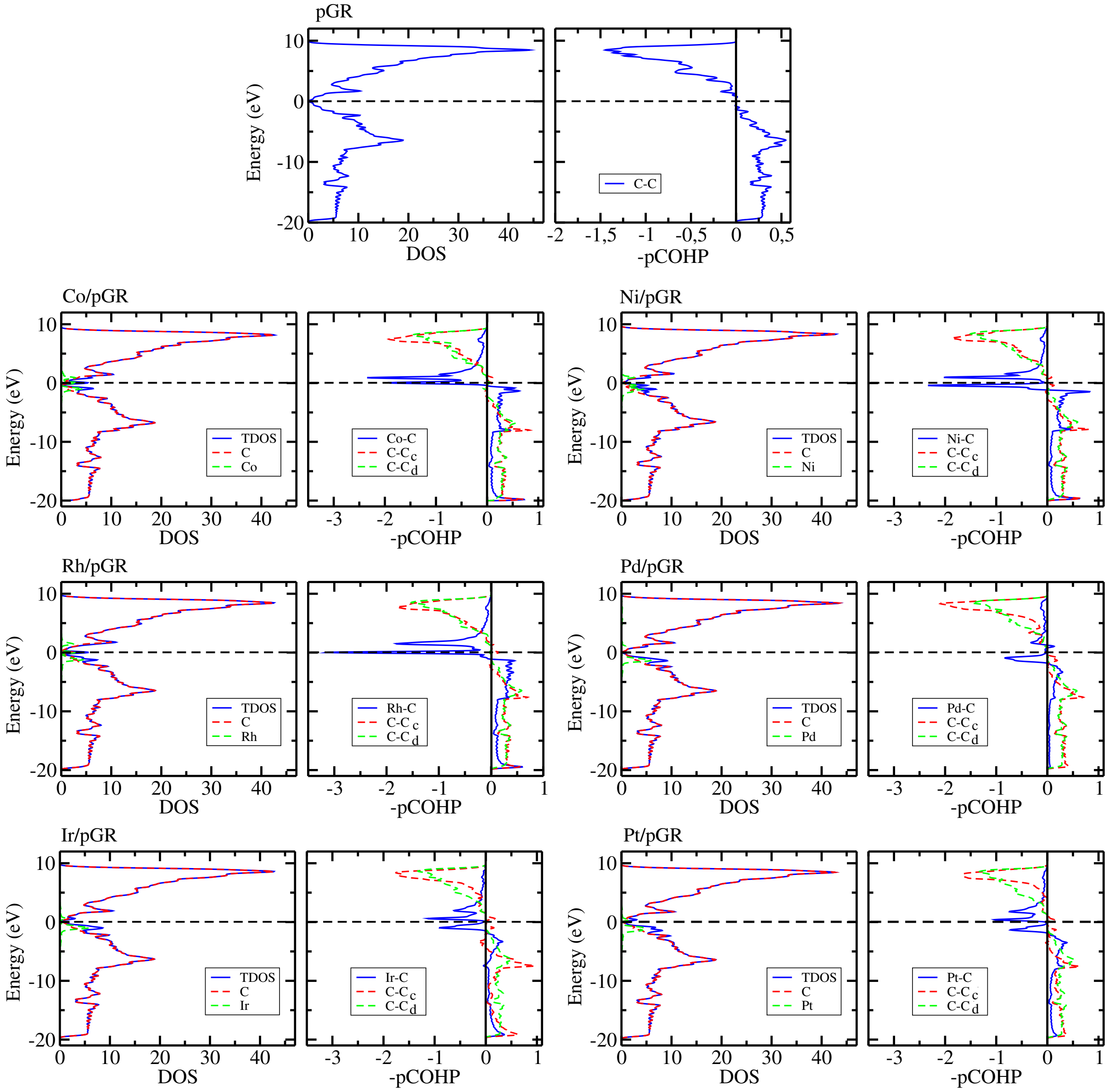

Figure S5: DOS and -pCOHP of pGR and TM/pGR. In the legends, "c" corresponds to the bond between close atoms, and "d" corresponds to the bond between distant atoms from the TM adsorption site. The dashed horizontal line indicates the Fermi energy, located at 0 eV.

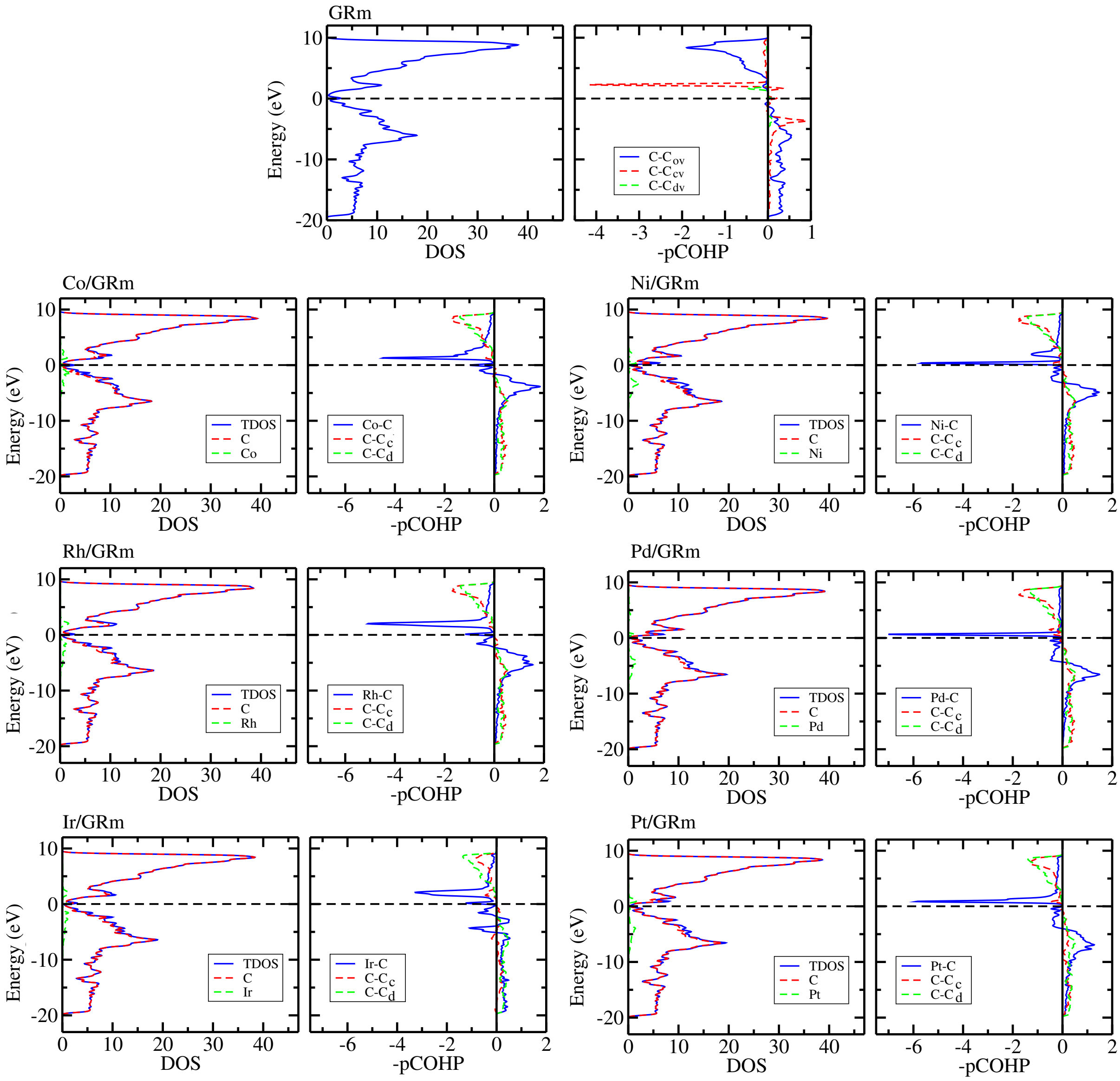

Figure S6: DOS and -pCOHP of GRm and TM/GRm. In the legends, "c" corresponds to the bond between close atoms, and "d" corresponds to the bond between distant atoms from the TM adsorption site. The dashed horizontal line indicates the Fermi energy, located at 0 eV.

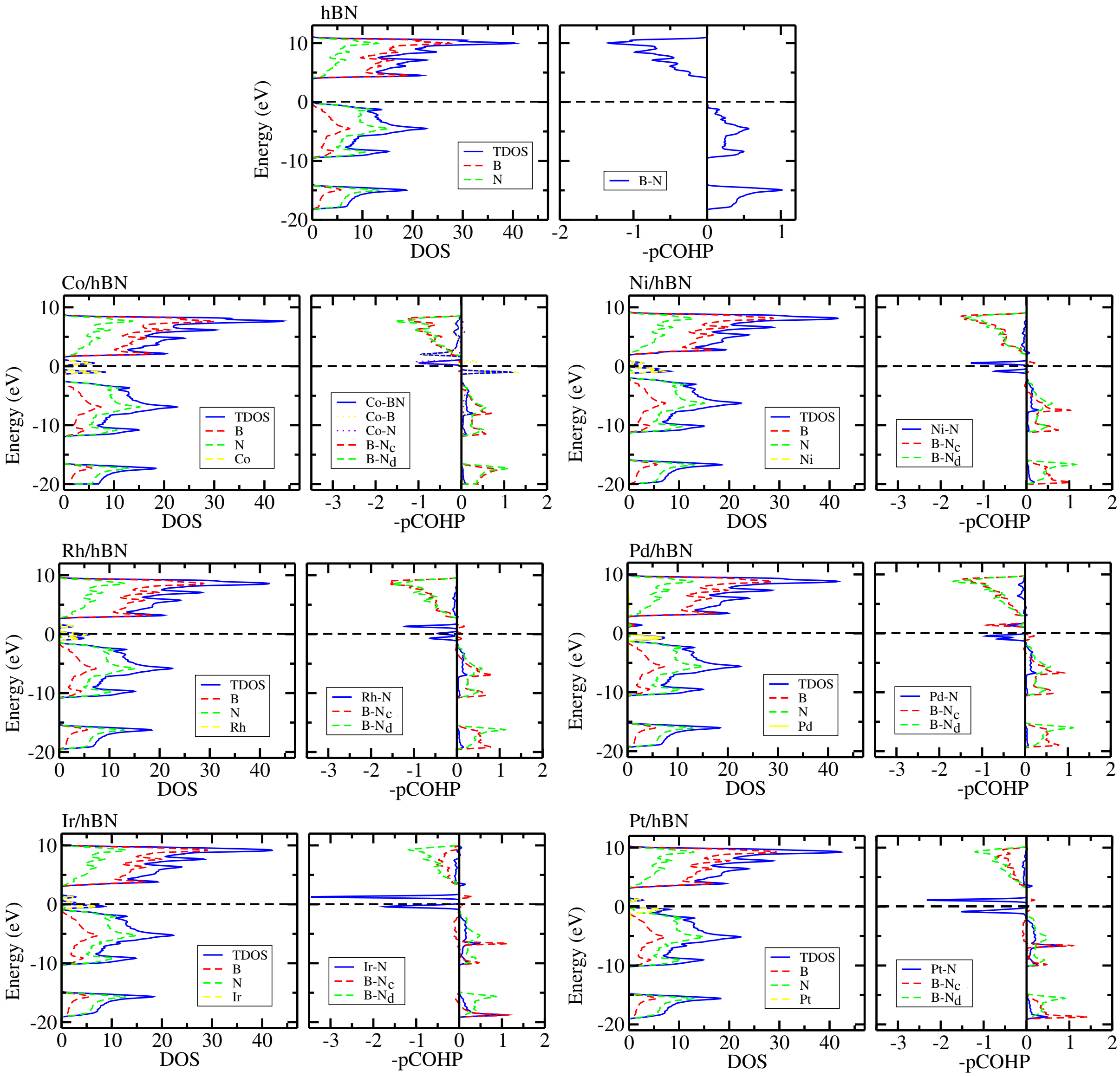

Figure S7: DOS and -pCOHP of hBN and TM/hBN. In the legends, "c" corresponds to the bond between close atoms, and "d" corresponds to the bond between distant atoms from the TM adsorption site. The dashed horizontal line indicates the Fermi energy, located at 0 eV.

5 Combined Systems - Bader Charge Analysis

Table S11: Charge distribution of the atoms constituting the systems: pGR, hBN, GRm, and their combinations with TM adsorption. The charge of the atom or the average charge of the C, B, and N atoms directly involved in TM adsorption (C_a, B_a, and N_a), the average charge of the C, B, and N atoms not directly involved in TM adsorption (C_b, B_b, and N_b), the charge of each atom directly involved in TM adsorption (C_{a_n}, B_{a_n}, and N_{a_n}), and the charge of the TM.

				pGR,	Q_{eff}	= 0.0 e			
	C_a	C_{a_1}	C_{a_2}	C_{a_3}	C_{a_4}	C_{a_5}	C_{a_6}	C_b	TM
Co	0.118	0.086	0.211	0.062	0.059	0.236	0.055	$-1.9\cdot10^{-3}$	-0.582
Ni	0.081	-0.027	0.073	0.198	0.202	-0.041	0.080	$-2.6\cdot10^{-4}$	-0.467
Rh	0.055	-0.040	0.043	0.171	0.174	-0.068	0.053	$-5.8\cdot10^{-4}$	-0.294
Pd	0.046	-0.030	0.121	-	-	-	-	$1.3\cdot10^{-3}$	-0.182
Ir	0.038	0.092	-0.016	-	-	-	-	$-5.5\cdot10^{-5}$	-0.072
Pt	-0.004	0.062	-0.071	-	-	-	-	$2.6\cdot10^{-4}$	-0.010
				hBN,	Q_{eff}	= 0.0 e			
	B_a	B_{a_1} | B_{a_2} | B_{a_3}		B_b	N_a	N_{a_1} | N_{a_2} | N_{a_3}		N_b	TM
Co	-2.132	-2.136 | -2.129 | -2.130		-2.183	2.180	2.180 | 2.179 | 2.180		2.182	-0.118
Ni	-	-		-2.173	2.173	-		2.180	-0.189
Rh	-	-		-2.175	2.096	-		2.181	-0.113
Pd	-	-		-2.176	2.090	-		2.180	-0.049
Ir	-	-		-2.175	2.028	-		2.179	0.026
Pt	-	-		-2.177	2.006	-		2.180	0.061
				GRm,	Q_{eff}	= 0.0 e			
		C_a	C_{a_1}	C_{a_2}	C_{a_3}	C_b		TM	
Co		0.203	0.205	0.195	0.207	$-4.7\cdot10^{-4}$		-0.576	
Ni		0.195	0.224	0.149	0.214	$-1.2\cdot10^{-3}$		-0.504	
Rh		0.124	0.138	0.117	0.116	$4.1\cdot10^{-4}$		-0.399	
Pd		0.166	0.154	0.177	0.166	$-2.0\cdot10^{-3}$		-0.360	
Ir		0.109	0.106	0.175	0.045	$1.0\cdot10^{-3}$		-0.394	
Pt		0.092	0.075	0.142	0.058	$1.5\cdot10^{-4}$		-0.285	
